# Synthesis, characterization, and theoretical study of new cocrystals and charge-transfer compounds

**DOI:** 10.55730/1300-0527.3766

**Published:** 2025-08-19

**Authors:** Zarife Sibel ŞAHİN, Zeki KARTAL

**Affiliations:** 1 Department of Energy Systems Engineering, Faculty of Engineering and Architecture, Sinop University, Sinop, Turkiye; 2 Retired Professor of Atomic and Molecular Physics, Kütahya Dumlupınar University, Kütahya, Turkiye

**Keywords:** 4-cyanopyridine (4CP), suberic acid (SA), 4-aminopyridine (4AP), isothiocyanate ion (NCS)^−^, tetracyanonickelate ion [Ni(CN)_4_]^2−^, Hirshfeld surface analysis

## Abstract

This study used single-crystal X-ray diffraction, elemental analysis, infrared (IR) spectroscopy, theoretical nuclear magnetic resonance (NMR), and theoretical ultraviolet spectroscopy to characterize 3 newly synthesized crystalline compounds. Additionally, the nonlinear optical, highest occupied molecular orbital energies, lowest occupied molecular orbital energies, band gap, molecular electrostatic potential, and thermodynamic parameters of the 3 crystalline compounds were examined. The strong correlation between experimental IR spectra and theoretical NMR chemical shifts confirmed the accuracy of computational predictions. The molecular formulas of the 3 newly synthesized crystalline compounds, each containing different ligand molecules, were: C_8_H_14_O_4_·2(C_6_H_4_N_2_), C_5_H_7_N_2_·NCS, and Ni(CN)_4_·2(C_5_H_7_N_2_)·2(H_2_O) for compounds 1, 2, and 3, respectively. Crystallographic analysis showed that the compounds crystallize in the space groups *P*1̄, *P*2_1_/*n* and *C*2/*m*, respectively. Their molecular packing is stabilized by a network of hydrogen bonds (C–H···O, O–H···N, N–H···N, N–H···S, O–H···N, and N–H···O) and noncovalent interactions (C–H···μ and μ···μ). Computational studies using Gaussian 03 and CrystalExplorer further elucidated their structural, magnetic, electrooptic, and electrochemical properties.

## 1. Introduction

Crystalline materials are fundamental to many scientific and industrial applications due to their well-ordered atomic structures, giving them unique physical and chemical properties. Depending on environmental conditions such as temperature, pressure, and concentration, atoms and molecules can form either crystalline or amorphous solids. Among these, crystalline structures have long-range order and diverse intermolecular interactions that significantly influence their optoelectronic and electrochemical behaviors [1,2].

Each component of a crystal compound has certain optoelectronic or electrochemical properties in their isolated form. When these components come together to form a crystalline compound, the properties of the new crystalline compound can take on very different values from those of their individual components [3]. Early on, scientists studied spontaneously formed crystals in nature and investigated some of their properties, leading to novel applications for crystals. Later, researchers began to create new crystals under controlled laboratory conditions. Recent advancements have enabled the synthesis of novel crystalline compounds with tailored properties. The ability to manipulate molecular orientation and composition has made it possible to develop crystals for specific functions such as gas storage, selective adsorption, and energy transfer [4].

Our previous studies have focused on the synthesis of Hofmann-type compounds and their clathrates using a variety of ligands and transition metals [5–23]. The ligand molecules we used in our previous studies can also be used to obtain novel crystal structures. These crystal structures were synthesized for use in energy production, safe energy transportation, and separation of various gas mixtures.

The ligands used in this study were 4-cyanopyridine (4CP), suberic acid (SA) (also known as octanedioic acid), 4-aminopyridine (4AP), potassium thiocyanate (KSCN), and potassium tetracyanonickelate monohydrate [K_2_Ni(CN)_4_.H_2_O]. The uncharged chemical compound 4CP (C_6_H_4_N_2_) has 2 nitrogen atoms in its structure. Due to the electron donating properties of the nitrogen atoms in both the pyridine ring and the cyan group, the 4CP ligand molecule can be used to obtain many polymeric structures. It can even cause the formation of 1D, 2D, and 3D metal–organic structures by bonding with the same or different transition metal atoms. However, the chemically active 4CP ligand molecule has not been examined in detail before. Compared to other ligand molecules, fewer resources are available for 4CP in the literature [24,25].

SA (C_8_O_4_H_14_) is a colorless, aliphatic compound with 2 carboxylic acid groups in its molecular structure. SA is a chemical substance produced in the bodies of plants, humans, and animals. SA is a very valuable compound with antiaging properties for the skin. It is used in the production of various drugs and in the industrial production of plastic materials [26–30].

4AP (C_5_H_6_N_2_) features an amino group positioned para to the nitrogen atom on a pyridine ring. This compound and its derivatives are commonly used in the development of novel chemical substances and in the treatment of various medical conditions [19,20,22].

Potassium thiocyanate (KSCN) is a fundamental compound used to generate thiocyanate ions (NCS^−^ or SCN^−^). These negatively charged, linear triatomic ions are monovalent and can participate in bonding through the carbon, sulfur, or nitrogen atoms, as well as via their μ-electron system in various chemical processes [31,32].

The compound K_2_Ni(CN)_4_.H_2_O is a yellow, water-soluble, diamagnetic compound. The structure of this compound consists of [Ni(CN)_4_]^2−^ anions and K^+^ cations. The [Ni(CN)_4_]^2−^ anions have a square planar structure. This compound plays a major role in obtaining new coordination compounds with very different structures [5–23].

This study aimed to systematically investigate and compare the various experimental and theoretical properties of crystalline substances formed by some of the aforementioned ligands, with a focus on their optoelectronic, structural, and electrochemical characteristics. To examine the precise molecular arrangements of the synthesized crystals, single-crystal X-ray diffraction (SC-XRD) was used as a primary structural characterization method. The results offer important insights into the potential uses of these new crystalline materials in diverse fields such as material science, medicine, and nanotechnology.

## 2. Experimental

### 2.1. Materials

To synthesize the desired compounds, the following reagents were used: 4CP (99%) (Fluka, Seelze, Germany), SA (98%) (Alfa Aesar, Haverhill, MA, USA), 4AP (Sigma-Aldrich, St. Louis, MO, USA), KSCN (96%) (Fluka), K_2_Ni(CN)_4_.H_2_O (96%) (Fluka), and aqueous ammonia solution (25%) (NH_3_, Merck, Darmstadt, Germany).

### 2.2. Synthesis of compounds

New crystalline compounds were synthesized through a series of chemical procedures. For compound 1, approximately 2 mmol 4CP (0.209 g) and approximately 1 mmol SA (0.102 g) were dissolved separately in a mixture of equal amounts of ethanol and water at 50 °C using a magnetic stirrer. One of these solutions was added dropwise into the other while constantly stirring. The entire mixture was stirred rapidly for 45 min at the same constant temperature. To make the entire mixture clearer, an aqueous ammonia solution was added dropwise. Finally, the entire mixture was stirred rapidly for 45 min at the same constant temperature, then filtered and allowed to crystallize.

For compound 2, approximately 1 mmol 4AP (0.095 g) and approximately 1 mmol KSCN (0.098 g) were dissolved separately in a mixture of equal amounts of ethanol and water at 50 °C using a magnetic stirrer. One of these solutions was added dropwise into the other while constantly stirring. The entire mixture was stirred rapidly for 45 min at the same constant temperature. To make the entire mixture clearer, an aqueous ammonia solution was added dropwise. Finally, the entire mixture was stirred rapidly for 45 min at the same constant temperature, then filtered and allowed to crystallize.

For compound 3, approximately 2 mmol 4AP (0.190 g) and approximately 1 mmol K_2_Ni(CN)_4_.H_2_O (0.259 g) were dissolved separately in a mixture of equal amounts of ethanol and water at 50 °C with the help of a magnetic stirrer. One of these solutions was added dropwise to the other while continuously stirred. The entire mixture was stirred rapidly for 45 min at the same constant temperature. To clarify the reaction medium, aqueous ammonia was introduced gradually into the mixture. The solution was then vigorously stirred for an additional 45 min while maintaining a constant temperature. It was then filtered and left under ambient conditions to allow crystallization. Within approximately 3 weeks, colorless transparent crystals formed for the first 2 compounds, while the third compound yielded a yellow, transparent crystal.

To ensure the charge balance of the newly synthesized compounds, the structure of compound 1 was expected to consist of one SA molecule and 2 4CP molecules. In order to balance the charges of the negatively charged NCS and Ni(CN)_4_ ionic ligands in compounds 2 and 3, any nitrogen atoms of the 4AP molecules were either protonated or the K^+^ ion in the environment was incorporated into the crystal structure of compounds.

### 2.3. Instrumentation

The Fourier transform-infrared (FT-IR) spectra of the synthesized crystalline materials were obtained using a Bruker (Billerica, MA, USA) Optics Vertex 70 FT-IR spectrometer. Elemental analysis was performed to assess carbon, nitrogen, hydrogen, and metal content using a Perkin-Elmer (Waltham, MA, USA) optima 4300 DV ICP-OES and CHNS-932 (LECO) analyzer.

Table S1 presents both experimental measurements and theoretical calculations of elemental composition for the crystalline compounds. The observed and predicted values for each newly synthesized compound show strong agreement, indicating consistency between theory and analysis.

From the SC-XRD experimental results of the compounds, the nitrogen atom of the pyridine rings of the 4AP ligand molecules in compounds 2 and 3 receives one H^+^ ion from the reaction medium. For this to occur, the solvent water molecules in the environment must interact with the K^+^ ion and ionize as H^+^ and OH^−^. The H^+^ ion binds to the nitrogen atom in the pyridine ring, while the OH^−^ ion combines with the K^+^ ion to form the basic substance KOH.

Suitable single crystals of each compound were chosen for structural analysis on a Bruker diffractometer using graphite-monochromated Mo-*K*_α_ radiation at 296 K. The structure solutions were obtained via direct methods using SHELXS-2013 software [33] and refined by full-matrix least squares techniques with SHELXL-2013 [34]. Data were collected using Bruker APEX2 software [35], molecular structures were visualized with Mercury [36], and additional processing was performed with WinGX [37].

The SC-XRD results and elemental analysis of their structures showed that the molecular formulas for crystalline compounds 1, 2, and 3, were C_8_H_14_O_4_·2(C_6_H_4_N_2_), C_5_H_7_N_2_·NCS, and Ni(CN)_4_.2(C_5_H_7_N_2_).2(H_2_O), respectively. Information regarding the data collection and crystal structure analysis for all compounds can be found in Table S2.

## 3. Results and discussion

### 3.1. Crystallographic analyses of compounds

#### 3.1.1. Crystallographic analysis of compound 1

**Figure 1:**
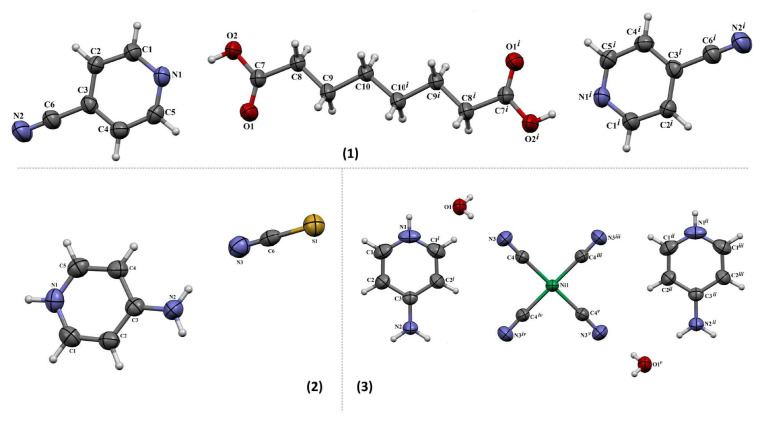
The molecular structures of compounds 1–3 (symmetry codes: *i*: −x, 2−y, 2−z for compound 1; *i*: x,1−y, z; *ii*: x, 1+y, z; *iii*: x, 2−y, z; *iv*: 1−x, y, 2−z for compound 2; *v*: 1−x, 2−y, 2−z for compound 3).

Figure 1 displays the atomic arrangement of compound 1, including the numbering pattern assigned to its atoms. The asymmetric unit of compound 1 consists of one 4CP and half a SA molecule. Compound 1 has a P1̄ space group. The C7–O1 bond distance [1.2038(18) Å] is typical of a double bond while the C6–N2 bond distance [1.140(2) Å] is typical of a triple bond. The O2–C7–C8–C9 torsion angle is −176.71(14)° (Table 1).

The molecules of compound 1 are linked by C–H···O and O–H···N hydrogen bonds (Table 2). The C4 atom in the molecule at (x, y, z) serves as a hydrogen-bond donor to the O1^i^ atom in the molecule at (−x+1, −y+1, −z+1), while the O2 atom in the molecule at (x, y, z) acts as a hydrogen-bond donor to the N1 atom in the same molecule, thereby forming centrosymmetric $R_{4}^{4}(16)$ rings that run parallel to the [111] direction (Figure S1). A strong hydrogen bond exists between the carboxylic group of the SA molecule and the nitrogen atom of the pyridine ring in the 4CP molecule. Similar interactions have also been documented in previous studies [28], particularly in the presence of the SA ligand. Both molecules incorporate a pyridine ring, further contributing to their structural resemblance.

Compound 1 also contains C–H···μ and μ···μ interactions. Information regarding these interactions can be found in Table 2.

#### 3.1.2. Crystallographic analysis of compound 2

Figure 1 shows the molecular structure of compound 2, with the corresponding atom labeling system. The asymmetric unit of this compound contains one 4-aminopyridin-1-ium (4APH) molecule and one isothiocyanate anion (NCS)^−^. For charge neutralization of compound 2, the N1 atom of the 4AP molecule bonds with the H^+^ ion in the environment and turns into a 4-aminopyridine-1-ium (4APH) molecule. Compound 2 has a *P*2_1_/*n* space group. The chosen geometric parameters are shown in Table 1.

Compound 2 features N–H···N and N–H···S hydrogen bonds (Table 2). As a result, $R_{8}^{6}(28)$ and $R_{4}^{4}(20)$ ring motifs are formed (Figure S2). Similar ring formations have also been observed in previous studies. An example is a compound formed by 2AP and citric acid (CA) molecules [4].

#### 3.1.3. Crystallographic analysis of compound 3

In compound 3, the asymmetric unit is composed of a quarter Ni(II) ion, half a 4APH molecule, one cyanide ion, and half a water molecule acting as a ligand (Figure 1). As in compound 2, the N1 atom in the 4AP molecule reacts with a free H^+^ ion present in the surrounding environment, transforming into the 4APH species and neutralizing the charge. The Ni(II) ion is bonded to 4 carbon atoms [Ni1–C4 = 1.8631(19) Å] from cyanide ligands, forming a square planar coordination geometry. As illustrated in Figure S3 and detailed in Table 2, the Ni(CN)_4_ clusters and 4APH molecules are joined by O–H···N and N–H···O hydrogen bonding interactions. These robust hydrogen bonds are crucial in assembling the extended 3-dimensional supramolecular architecture.

There were notable differences in the crystal structures of the 3 compounds. Compound 1 crystallized in the triclinic *P*1̄ space group and had C–H···O and O–H···N hydrogen bonding between neutral organic ligands (4CP and SA). In contrast, compound 2 adopted a monoclinic *P2_1_/n* space group, containing ionic species such as 4APH^+^ and NCS^−^ stabilized by N–H···N and N–H···S hydrogen bonds. Compound 3, also monoclinic (C2/m), incorporates a transition metal coordination complex, where Ni^2+^ is coordinated to 4 cyanide ligands in a square planar geometry, forming metal-ligand frameworks along with water and protonated 4AP ligands. The presence of a metal center and coordinated solvent molecules in compound 3 makes its supramolecular architecture more complex and distinct from the purely organic or semiionic systems in compounds 1 and 2. Furthermore, the symmetry and hydrogen bonding motifs differ significantly across the compounds, resulting in variations in molecular packing, crystal voids, and overall lattice stability.

### 3.2. Experimental FT-IR studies of compounds

**Figure 2:**
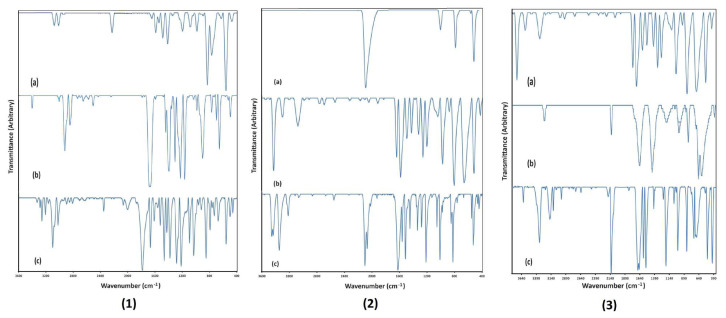
The FT-IR spectra of free ligand molecules 4CP (1a), SA (1b), and compound 1 (1c); KNCS (2a), 4AP (2b), and compound 2 (2c); and 4AP (3a), K_2_Ni(CN)_4_.H_2_O (3b), and compound 3 (3c).

Since some of the ligand molecules in compounds are different, each of the compounds has its own unique spectroscopic properties. Since the most dominant interactions during the formation of the crystal structures are weaker interactions such as H···H, N···H, O···H, S···H, and C···H, and they do not contain any metal bonds in their structures, the FT-IR spectra of these compounds were very close to the sum of the FT-IR spectra of the free state of the constituent ligand molecules. Herein, the FT-IR spectra of the free state ligand molecules that form each compound and the FT-IR spectrum of the resulting compound will be given together (Figure 2) and the results are shown comparatively in Table S3.

#### 3.2.1 FT-IR studies of compound 1

There are 2 different molecular structures in the structure of compound 1, namely 4CP and SA ligand molecules (Figure 1). The 4CP molecules is aromatic and SA is aliphatic. The physical, chemical, and structural properties of these molecules are formed by the groups they have, such as C=N–C, C≡N, COOH, and C–H.

The 4CP ligand molecule is a planar aromatic, uncharged compound with C*_2v_* point group formed by the nitrile group bonded in the para position to the nitrogen atom of a pyridine ring consisting of a total of 12 atoms. Therefore, the 4CP molecule has 30 vibration modes and almost all of them are IR active modes [38,39]. When the 4CP molecule participates in the formation of a compound, some of these modes may shift to high frequency and some to low frequency due to the bonds it makes or environmental changes. Similar situations apply to every molecule participating in the formation of a compound.

The SA ligand molecule, is a dicarboxylic acid with a closed formula C_8_H_14_O_4_, consisting of 26 atoms in an aliphatic structure. The space group of the crystal structure of the SA molecule is P2_1_/c and there are 2 SA molecules in each unit cell [40]. In the vibration spectroscopy of SA, 72 vibration modes are expected to occur. However, since its structure has high symmetry, fewer vibration modes were observed experimentally. FT-IR spectra of the free 4CP and SA ligand molecules and compound 1 are shown in Figure 2.

Some vibration modes of the 4CP and SA ligand molecules remain largely uncharged or are only minimally altered by the assembly of the crystal structure. These modes are ν(C≡N), ν(C=C), ν(C–C), and ν(C–N) for the 4CP ligand molecule, while they are ν(C–H) aliphatic, ν(COH), δ(CH_2_), δ(C=O), δ(C–O), and ω(CH_2_) for the SA ligand molecule.

As shown in Figure S1, 2 aromatic hydrogen atoms of the 4CP ligand engage in interactions with the oxygen atoms of the SA ligand in compound 1, while the other 2 hydrogen atoms remain uninvolved. Accordingly, there are 2 ring hydrogen groups with different properties. Therefore, the ν(C–H) stretching vibrations in the FT-IR spectrum of compound 1 are split into two. In addition, these vibration modes are shifted to higher wavenumbers, such as 18–74 cm^−1^, compared to their free state (Table S3). Any functional group will shift its vibration mode to higher wavenumbers due to its participation in the formation of a new bond [4,15,28].

Some vibration frequencies in the SA ligand for compound 1 were substantially altered due to the creation of new bonds that form the crystal structure. The ν(O–H), δ(O–H), and ν(C=O) modes of the carboxylic group are examples of these vibration modes. Figure S1 shows that the vibration states of the SA ligand molecule are involved in the interactions that play a role in the construction of compound 1. The ν(O–H) vibration mode of the SA ligand molecule in the crystal structure of compound 1 shifted to a low wavenumber of 748 cm^−1^ due to the interaction of the hydrogen atom in this structure with the ring nitrogen of the 4CP ligand molecule. Due to the same interaction, the δ(O–H) vibration mode shifted to a high wavenumber of 235 cm^−1^. Such high or low wavenumber shifts have also been observed in previous studies [41–43].

The ν(C=O) vibration mode of the SA ligand molecule in the crystal structure of compound 1 shifted to a high wavenumber of 26 cm^−1^ due to the interaction of the oxygen atom in this structure with the hydrogen atom of the 4CP ligand molecule. The extent of the shift toward higher wavenumbers observed in this vibration mode is much smaller than the shifts occurring in other modes. In addition, due to the same interaction, shifts towards wavenumbers as low as 11 and 14 cm^−1^ were observed in the δ(C=O) vibration modes (Table S3).

#### 3.2.2 FT-IR studies of compound 2

Two different ligand molecules formed compound 2: 4AP and the KSCN ligand molecules. The FT-IR spectra of the free 4AP and KSCN ligand molecules and compound 2 are shown in Figure 2. The first of these ligand molecules has an aromatic structure while the second has a linear structure. The physical, chemical, and structural properties of these ligand molecules are determined by the C=N–C, C–H, NH_2_, C≡N, and C–S groups found in their structures.

The 4AP ligand molecule, present in the structure of both compounds 2 and 3, is an aromatic, uncharged compound with a planar structure formed by the NH_2_ group attached to the nitrogen atom of a pyridine ring in the para position, with the closed formula C_5_H_6_N_2_. More information about the 4AP ligand molecule is detailed in the literature [19,20,22,44,45]. If the 4AP ligand molecule also participates in the formation of a compound, some of its vibration modes may shift to higher or lower wavenumbers due to the bonds it forms with other atoms and changes in environmental conditions.

As shown in Figure S2, the nitrogen atom in the aromatic ring of the 4AP ligand molecule in the crystal structure of compound 2 has taken a hydrogen atom from the reaction medium to ensure the charge balance in the compound. As a result of this newly formed N–H bond, a new ν(N–H) stretching peak at 3437 cm^−1^ wavenumbers appeared in the FT-IR spectrum of compound 2 (Table S3). Similar results have been reported in other studies of ligands with pyridine rings [46–48].

As shown in Table S3, the NH_2_ stretching mode of the 4AP ligand molecule in compound 2 undergoes a shift to higher wavenumbers, with the ν_as_(NH_2_) and ν_s_(NH_2_) stretching frequencies increasing by 28 and 46 cm^−1^, respectively. Such shifts have also been noted in previous studies by ourselves and other researchers [19,20,22,44,45].

Similarly, one of the vibration modes of the KSCN ligand molecule affected by the formation of compound 2 is ν(C≡N). This vibration mode was divided into three due to the interaction applied to the C≡N group by the other groups at 3 different values. Two of them shifted to high wavenumbers (60 and 35 cm^−1^) and one shifted to a lower wavenumber (13 cm^−1^) (Table S3). Similar shifts have also been reported in other studies [49].

The two vibration modes of the KSCN ligand molecule that are most affected by the formation of compound 2 are the ν(C–S) stretching mode and the δ(NCS) bending mode. These vibration modes are also shifted to wavenumbers as high as 84 and 55 cm^−1^, respectively.

#### 3.2.3 FT-IR studies of compound 3

Three different ligand molecules—4AP, H_2_O, and K_2_Ni(CN)_4_·H_2_O—were involved in the formation of compound 3. The FT-IR spectra of the free 4AP and K_2_Ni(CN)_4_·H_2_O ligand molecules and compound 3 are shown in Figure 2. Of these ligand molecules, 4AP has an aromatic planar structure, H_2_O has an angled structure, and K_2_Ni(CN)_4_ has a square planar structure with Ni atoms in the center and CN groups at the corners. The physical, chemical, and structural properties of these ligand molecules are determined by the charged or uncharged C=N–C, C–H, N–H, NH_2_, O–H, and C≡N groups in their structures.

As shown in Figure 1, the nitrogen atom in the aromatic ring of the 4AP ligand molecule in the crystal structure of compound 3 made a single bond with a hydrogen atom from the reaction medium to ensure charge balance in this compound. This new single bond created a strong stretching vibration at a wavenumber of 3334 cm^−1^ in the FT-IR spectrum of compound 3 (Table S3).

## 4. Computational studies of compounds

The structural parameters of all crystalline compounds were calculated in the Gaussian 03 program [50] using the DFT/B3LYP method [51–54] and the 6-311G(d,p) basis set. According to experimental data, there is an Ni transition metal atom in the structure of compound 3. Therefore, the structural parameters of compound 3 were also calculated in the Gaussian 03 program [50] using the DFT/B3LYP method [51–54] on the LanL2MB basis set. The LanL2MB basis set gives more consistent results than other basis sets in compounds containing transition metals in their structures. Thus, all compounds were compared with the values obtained with the same basis set. Theoretical data were used to estimate various electronic, magnetic, and thermochemical characteristics of the synthesized compounds [50]. Visualization of the computed results for all compounds was carried out using GaussView version 4.1 software [55].

### 4.1. Mulliken atomic charge values of ligands and compounds

**Figure 3:**
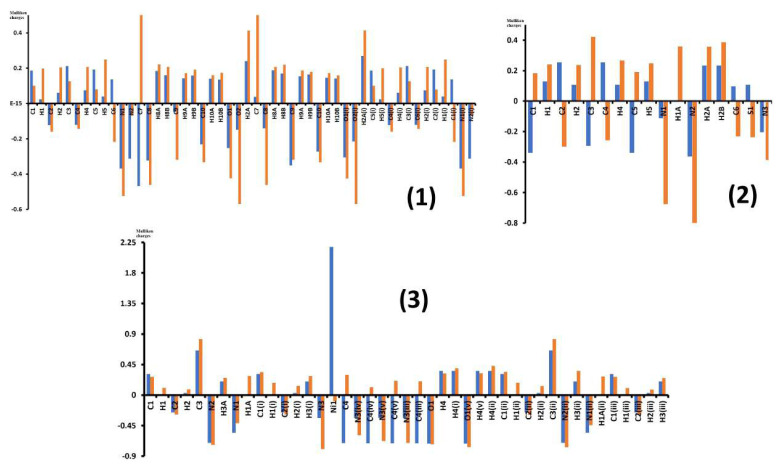
Mulliken charges are illustrated for compounds 1–3, with blue indicating values in the uncoordinated ligand state and red showing those within the coordinated compound structure.

The electric charges present on each atom of an electrically balanced compound are called Mulliken charges. The Mulliken charge values and locations determine the electrical and magnetic properties of a compound. To facilitate comparison, the Mulliken charge values of the atoms in the free ligands and the atoms in the compounds are given in Table S4 and Figure 3 in terms of free electron charge (e).

In Figure 3, the first bar (blue color) in the cell corresponding to each atom shows the electrical charge value of that atom before forming the compound. The second bar (red color) shows the electrical charge value of that atom in the compound.

The following findings are evident in Table S4 and Figure 3:

- The charge values of atoms changed after compound formation compared to their free ligand states.
- In the formation of compounds, the electrical charge sign of some atoms remains the same and only their charge value changes. These atoms are in compound 1: C1, H1, and C2; compound 2: H1, H2, H4 H5, N1, and N2; and in compound 3: C1, C2, C3, and N2.
- Due to the formation of compounds, both the electric charge value and the electric charge sign of some atoms have changed. These atoms are in compound 1: C6 and C7; compound 2: C1, C2, C3, C4, C5, C6, and S1; and compound 3: Ni1, C4, C4^i^, C4^ii^, and C4^iii^.
- Due to the formation of compounds, the greatest change in the electrical charge values are in compound 1: C7, O2, C7^i^, C8^i^, and O2^i^; compound 2: C1, C2, C3, N1, and N2; and in compound 3: Ni1, C4, C4^iii^, C4^iv^, and C4^v^.
- The H1A atom in compounds 2 and 3 do not belong to the ligand molecules that form these compounds. These hydrogen atoms were added to the compounds from the reaction medium.

### 4.2. HOMO–LUMO energy levels of compounds

Each atom has atomic orbitals that correspond to certain energy levels, and generally where electrons are most likely to be located. When various atoms form a compound by interacting with each other, these atomic orbitals fuse with each other to form molecular orbitals.

Within a molecule, orbitals are either fully or partially occupied by electrons while some remain vacant. The energy levels of the highest occupied molecular orbital (HOMO) and the lowest unoccupied molecular orbital (LUMO) play a crucial role in defining the chemical and physical behavior of a compound. Moreover, the energy gap between HOMO and LUMO influence the stability and optical properties of the compound in solution, including their color.

**Figure 4:**
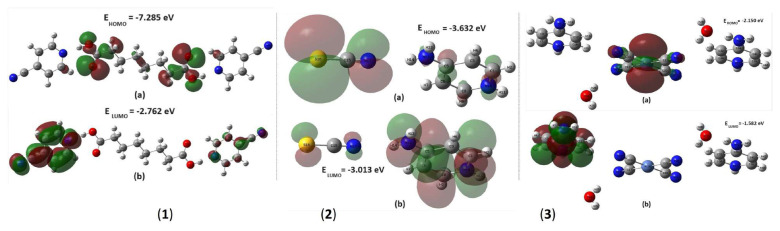
The HOMO and LUMO energy graphs of compounds 1–3.

Figure 4 shows the HOMO and LUMO energy diagrams of the compounds obtained though theoretical calculations. In order to compare the HOMO and LUMO energy values of all compounds with each other, the DFT/B3LYP approach with the 6-311G(d,p) basis set was used [51–54]. Since compound 3 contains an Ni atom in its structure, the DFT/B3LYP approach with the LanL2MB basis set was applied. Thus, HOMO and LUMO values were calculated using both 6-311G(d,p) and LanL2MB basis sets for compound 3 and compared to each other. The calculated ΔE values did not cause any change in the ranking of kinetic stabilities or chemical reactivities of all 3 compounds, despite the different values obtained for this compound (Table 3).

For compound 1, the HOMO distribution is predominantly localized on the carboxyl functional groups of the SA ligand, with a minor contribution observed on the C5–H5 and C2^i^–H1^i^ fragments of the 4CP ligands. In contrast, the LUMO of the same compound is fully localized on the 2 4CP ligands within its molecular framework (Figure 4). However, the 4CP ligand molecule on the right side of the figure has weaker LUMO values and the 4CP ligand molecule on the left side of the figure has stronger LUMO values.

The HOMO state of compound 2 occurs on the 2 nitrogen atoms of the 4AP ligand molecule; carbon atoms C1, C3, and C5 of the pyridine ring; and atoms S1 and N3 of the NCS ion. The LUMO state is distributed over the remaining regions of the 4AP ligand, excluding the C2 carbon and hydrogen atoms, while also involving the S1 and N3 atoms of the NCS ion (Figure 4).

The HOMO state of compound 3, calculated using the specified basis set, is primarily located on the Ni atom and Ni–C single bonds within the Ni(CN)_4_ ionic group. According to the LanL2MB basis set results, the LUMO state of the compound occurs entirely on the C and N atoms of the 4APH ionic ligand molecule on the left side of the structure (Figure 4).

Equations 1–5 were used to theoretically determine the electrochemical characteristics of the compounds based on their HOMO and LUMO energy levels.


(1) $$\Delta E=E_{LUMO}-E_{HOMO}\;(\text{Energy gap value})$$

(2) $$\chi =\frac{\left(I+A\right)}{2}=-\mu \;(\text{Electronegativity},\text{ Negative chemical potential})$$

(3) $$\eta =\frac{\left(I-A\right)}{2}(\text{Chemical hardness})$$

(4) $$S=\frac{1}{2\eta }(\text{Chemical softness})$$

(5) $$\omega =\frac{\mu^{2}}{2\eta }(\text{Electrophilicity index})$$

Table 3 displays the chemical efficiency values of the compounds, calculated using equations 1–5 based on their HOMO and LUMO energy levels.

The HOMO–LUMO energy gap (ΔE) decreases in the order: ΔE_1_ > ΔE_2_ > ΔE_3_. This trend suggests that compound 1 has relatively high kinetic stability and low chemical reactivity in comparison with compounds 2 and 3.

When comparing electronegativity values, the compounds follow the sequence χ_1_ > χ_2_ > χ_3_, and the chemical hardness values follow the sequence η_1_ > η_2_ > η_3_. Conversely, the chemical softness values are ranked as S_3_ > S_2_ > S_1_, and the electrophilic index values follow the trend ω_2_ > ω_3_ > ω_1_.

Furthermore, given that compound 3 had the highest chemical softness (S) and the lowest chemical hardness (η), the intramolecular charge transfer is expected to be more pronounced in this compound relative to the others.

### 4.3. Nonlinear optical properties of compounds

In molecules made up of multiple atoms, the electric dipole moment (*μ̄*) originates from the spatial distribution of electrical charges within the structure. This property is typically illustrated as a vector in 3-dimensional space. Variations in *μ̄* reflect internal shifts in charge positions throughout the molecule. The direction and magnitude of this dipole moment depend on the arrangement of positive and negative charge centers. When a molecule reaches electrostatic stability, its dipole moment remains unchanged, allowing its orientation to be clearly defined.

When an external electric field interacts with a molecule in electrostatic equilibrium, the internal charge distribution becomes distorted, leading to a shift in the balance of charges. The tendency of the electron cloud to become redistributed under an applied field is known as the polarizability of the compound. In most contexts, polarizability (α) is commonly used in place of average polarizability (α_0_).

Using the 6-311G(d,p) basis set within the DFT/B3LYP framework, μ, α_0_, Δα, β_0_, and γ [56–58] for compounds 1 and 2 were theoretically computed. The same values were computed for compound 3 using the LanL2MB basis set determined in DFT/B3LYP. Equations 6–13 were used to calculate the μ, α_0_, Δα, β_0_, and γ values of the compounds, respectively.


(6) $$\mu =\sqrt{\mu_{x}^{2}+\mu_{y}^{2}+\mu_{z}^{2}}\;(\text{Dipole moment})$$

(7) $$\alpha_{0}=\frac{\alpha_{xx}+\alpha_{yy}+\alpha_{zz}}{3}(\text{Mean polarizability})$$

(8) $$\Delta \alpha =\sqrt{\frac{{\left(\alpha_{xx}-\alpha_{yy}\right)}^{2}+{\left(\alpha_{yy}-\alpha_{zz}\right)}^{2}+{\left(\alpha_{zz}-\alpha_{xx}\right)}^{2}+6(\alpha_{xy}^{2}+\alpha_{xz}^{2}+\alpha_{zy}^{2})}{2}}(\text{Anisotropies of polarizability})$$

(9) $$\beta_{x}=\beta_{xxx}+\beta_{xyy}+\beta_{xzz}\;(\text{x component of }\beta_{0})$$

(10) $$\beta_{y}=\beta_{yyy}+\beta_{xxy}+\beta_{yzz}\;(\text{y component of }\beta_{0})$$

(11) $$\beta_{z}=\beta_{zzz}+\beta_{xxz}+\beta_{yyz}\;(\text{z component of }\beta_{0})$$

(12) $$\beta_{0}=\sqrt{\beta_{x}^{2}+\beta_{y}^{2}+\beta_{z}^{2}}\;(\text{First-order static hyperpolarizability})$$

(13) $$\gamma =\frac{1}{5}\left\{\gamma_{xxxx}+\gamma_{yyyy}+\gamma_{zzzz}+2[\gamma_{xxyy}+\gamma_{xxzz}+\gamma_{yyzz}]\right\}\;(\text{Second-order static hyperpolarizability})$$

The results are given in Table 4. Of these calculated values, α_0_, Δα, β0 and γ are given in Table 4 in electrostatic units (esu) using the necessary conversion factors [59,60]. The positive and negative charge centers of symmetric structures coincide. Therefore, the dipole moments of symmetric structures are zero. Compounds 1 and 3 have highly symmetrical parts. Therefore, their dipole moment values are small. However, compound 2 has the least symmetrical parts. Therefore, its dipole moment value is the largest compared to the other compounds.

Here is a summary of the findings in Table 4:

- Among all the compounds examined, compound 2 has the largest dipole moment (μ = 25.4790 D), highlighting its stronger molecular polarity and greater sensitivity to external electric influences.
- The sequence of polarizability anisotropy (Δα) values from highest to lowest is as follows: (Δα)_3_ > (Δα)_1_ > (Δα)_2_. Additionally, the relative magnitudes are: (Δα)_3_= 3.295 (Δα)_1_ = 8.575 (Δα)_2_, indicating that compound 3 undergoes the most significant directional variation in polarization.
- The descending order of average polarizability (α_0_) is: (α_0_)_2_> (α_0_)_1_ > (α_0_)_3_. In addition, the alpha values are: (α_0_)_2_ = 2.422 (α_0_)_1_ = 1.552 (α_0_)_3_. This indicates that compound 2 has the highest ability to polarize, with compounds 1 and 3 showing lower polarizability.
- The order of β_0_ values from largest to smallest is (β_0_)_2_ > (β_0_)_3_ > (β_0_)_1_. In addition, the β_0_ are: (β_0_)_2_ = 1.304 (β_0_)_3_ = 19.956(β_0_)_1_. In examining the nonlinear optical (NLO) properties of the compounds, the β_0_ value of urea was taken as a reference. The β_0_ values of the compounds are 1.30, 15.34, and 11.77 times greater than those of urea, respectively. Compound 2 had the greatest nonlinear response to electric fields.
- For second order hyperpolarizability (γ), the values decrease in the order: γ_1_ > γ_3_ > γ_2_. In addition, the γ values are: γ_1_ = 5.814γ_3_ = 13.204γ_2_. It suggests that compound 1 has the greatest second-order response compared to the other compounds.

In summary, compound 2 had the greatest values of μ, α_0_, and β_0_, indicating it has the strongest polarization and response to electric fields overall. Compound 3 had the highest directional polarizability variation, and compound 1 stands out for its second-order nonlinear response.

### 4.4. Thermodynamic parameters of compounds 1–3

The behavior of the compounds in response to heat under normal conditions and their thermochemical parameters are unknown and it is challenging to examine these parameters experimentally. Therefore, quantum chemical calculations are important to examine the thermodynamic properties of a compound. The theoretical thermochemical properties calculated for all 3 compounds are given in Table 5. The rankings of the compounds from largest to smallest according to the total values of their thermodynamic parameters are 1, 3, and 2 for E and E_v0_ values, and 3, 1, and 2 for C_v_ and S values.

### 4.5. Molecular electrostatic potential of compounds 1–3

**Figure 5:**
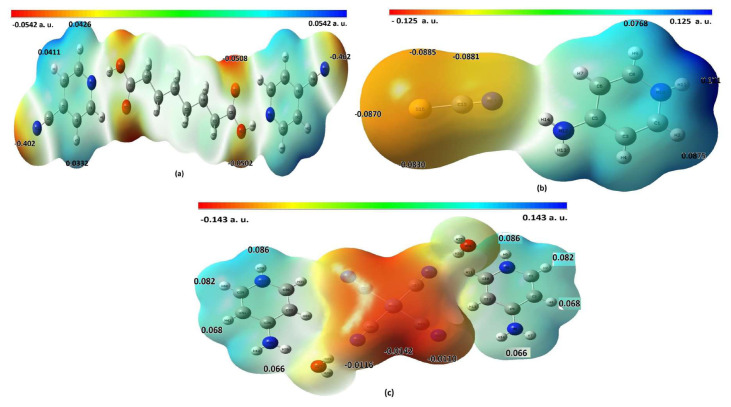
MEP maps of compounds 1 (a) and 2 (b) calculated according to DFT/B3LY/6-311G(d,p), and 3 (c) calculated according to DFT/LanL2DZ levels.

The molecular electrostatic potential (MEP) calculations of the compounds were performed with the B3LYP/6-311G(d,p) basis set for compounds 1 and 2, and with the B3LYP/LanL2DZ basis set for compound 3. Figure 5 shows the MEP maps of the relevant compounds. In the MEP map of each compound, the points marked in red and yellow indicate high electron density and negatively charged regions, while the parts marked in blue and white indicate low electron density and positively charged regions. In the MEP maps of the compounds, the negative (red and yellow) regions are associated with electrophilic reactivity, while the positive (blue and white) regions are associated with nucleophilic reactivity (Figure 5).

According to the MEP map in Figure 5a, the electronegativities of the nitrogen atoms in the CN groups and the oxygen atoms in the carbonyl groups of compound 1 are higher than the other atoms. For this reason, most of the electron density in compound 1 is located in these regions. In contrast, the dispersion regions of positive charges in compound 1 are concentrated in the hydrogen atoms bonded to the carbons in the pyridine ring and chain structure.

Similarly, the MEP map in Figure 5b shows the increased electronegativities of the C, N, and S atoms in the isothiocyanate group of compound 2. Therefore, the entire electron density in compound 2 is in the NCS ionic structure. In contrast, the distribution regions of positive charges in compound 2 are located entirely in the 4APH ionic ligand and increase further away from the NCS ionic group.

The MEP map in Figure 5c shows that the electron density of compound 3 is entirely located in the Ni(CN)_4_ ionic structure and partially in the water molecules. In contrast, the distribution regions of positive charges are entirely located in the 4APH ionic ligands and increase in regions farther from the Ni(CN)_4_ ionic group.

The MEP maps show that compounds 1 and 3 have very symmetric charge distributions, while compound 2 has almost no symmetric charge distribution. This is consistent with the dipole moment values of the compounds.

### 4.6. Hirshfeld surface analysis of compounds 1–3

**Figure 6:**
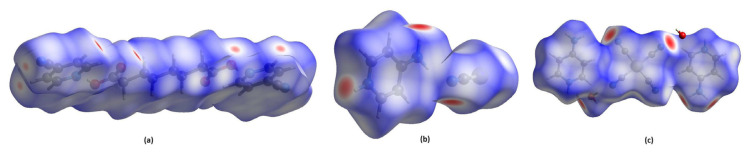
The d_norm_ views of the 3D Hirshfeld surfaces of the smallest units of the compounds from different viewing angles: compound 1 (a), compound 2 (b) and compound 3 (c).

In the formation of a crystal structure, metallic bonds and long-range interactions between various atoms are essential in addition to short-range interactions, including hydrogen bonding and van der Walls forces. The intensities of these short-range interactions are less than the intensities of other interactions. Spackman et al. [61] developed the CrystalExplorer program that uses cif files to calculate various molecular surface properties, including Hirshfeld surfaces, crystal vacancies, electron density, deformation density, electrostatic potentials, molecular orbitals, and spin density. Additionally, it produces a 2D representation of the 3D Hirshfeld surface, known as the fingerprint map of the compound. This provides valuable insights into the interactions between the closest adjacent atoms in the molecular structure [61]. Hirshfeld surface analysis identifies intermolecular interactions, with red, blue, and white regions representing different interaction types. The intensity of these colors reflects interaction strength. Red regions correspond to hydrogen bonds, blue regions indicate interactions effective over longer distances, and white regions show van der Waals interactions [61]. This method offers a graphical representation that makes it easier to interpret interactions between different molecules within a structure [61]. The d_norm_ views of the 3D Hirshfeld surfaces generated for all compounds using CrystalExplorer, along with their 2D fingerprint plots and the percentage contributions of various interactions, are presented in Figure 6, Figure S4, and Table 6.

The primary factor contributing to the formation of the crystal structures in all compounds is H···H interactions. The impact of these H···H bonds on the compounds follows the order: compound 1, compound 3, and compound 2. Additionally, the smallest contribution to the crystal formation is from the O···O interactions in compound 1, C···C interactions in compound 2, and C···Ni/Ni···C interactions in compound 3.

Figure S5 shows the shapes of the spaces formed in their 1 × 1 × 1 dimensional unit cells for each of the 3 compounds. The numerical values of these voids are obtained by adding the spherical electron densities around the appropriate atomic nuclei for each compound (Table S5). The void values of the compounds decrease in the order: compound 3, compound 2, and compound 1.

The ratio of void volume to the unit cell volume increase in the following order: compound 2, compound 1, and compound 3. Further investigation into the storage properties of these crystalline compounds for various gaseous substances under different conditions is necessary.

The structural integrity of the packing within a crystal can indicate how the crystal responds to external mechanical forces. When a crystal structure contains substantial voids, the molecules within it exert insufficient force on each other, leading to poor packing. As a result, such a structure may be prone to breaking under even minimal external mechanical stress. This makes the mechanical stability of crystal structures, particularly those intended for critical applications like gas storage, highly significant.

The molecular interaction energies resulting from the interactions between the components forming a crystal structure, and the sum of these interaction energies can also be calculated with the CrystalExplorer program.

The total molecular energy (in kjoule/mol) resulting from the interactions between the reference molecule in a crystal structure and other neighboring molecules consists of the sum of 4 different types of energy: electrostatic, polarization, dispersion, and exchange repulsion (Equation 14).


(14) $${\text{E}}_{\text{tot}}={\text{E}}_{\text{ele}}+{\text{E}}_{\text{pol}}+{\text{E}}_{\text{disp}}+{\text{E}}_{\text{rep}}$$

When calculating the values of E_tot_ for a molecular structure, CrystalExplorer uses the HF/3-21G basis set, which provides fast and simple calculations, or the B3LYP/6-31G(d,p) basis set, which is slower but more accurate. The B3LYP/6-31G(d,p) basis set was used to calculate E_tot_ and its components for the 3 compounds. The scale factors used for the B3LYP/6-31G(d,p) basis set were k_ele_ = 1.057, k_pol_ = 0.740, k_disp_ = 0.871, and k_rep_ = 0.618 [61].

Two methods were used to calculate the values of E_tot_ of the compounds. For compounds 1 and 3, the molecules at the center of symmetry were selected as reference molecules. The missing structures of other neighboring molecules located 3.8 Å from the center of these molecules were completed. In compound 2, the 4APH ligand molecule was selected as the reference molecule. The missing structures of other neighboring molecules located 3.8 Å from the center of this molecule were completed. E_tot_ corresponding to the specified conditions were calculated for each compound. The grouping of interacting molecules of each compound and the calculation result table of the values of E_tot_ are shown in Figure S6.

**Figure 7:**
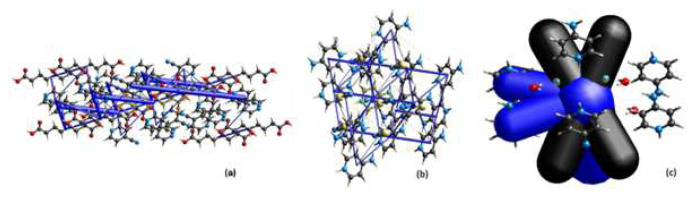
Graphs of E_tot_ values of compounds obtained under the same conditions. Compound 1 (a), compound 2 (b), and compound 3 (c).

E_tot_ values were −127.2, −67.3, and −236.1 kJ/mol for compounds 1, 2, and 3, respectively. The E_tot_ values for the compounds follow the descending order: compound 3 > compound 1 > compound 2. The accuracy of the order of magnitude of the E_tot_ values is shown in Figure 7.

### 4.7. Theoretical UV and visible spectra of compounds 1, 2 and 3

**Figure 8:**
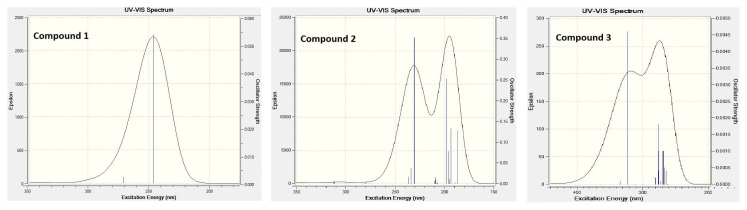
Theoretical UV–visible absorption spectra of compounds in water solvent.

The theoretical UV–visible light absorption spectra of compounds 1 and 2 were analyzed using the time-dependent self-consistent field (TD-SCF) with the B3LYP/6-311G(d,p) basis set [scrf = (iefpcm, solvent = water)] conditions, for N_state_ = 20. The theoretical UV–visible light absorption spectrum of compound 3 was analyzed using the B3LYP/LanL2DZ basis set under the same conditions. The UV–visible light data of the electronic transitions of the compounds were obtained by plotting the wavelengths (λ, in nanometers) of the electronic transitions against their oscillation strengths (f, unitless) (Figure 8) [62]. Table S6 provides examples of several electronic transitions observed in the compounds, along with their corresponding characteristics. Oscillator strength of the compounds varies between zero and one depending on whether an electronic transition in the UV–visible spectrum is forbidden or allowed (Table S6 and Figure 8).

According to Figure 8 and Table S6, compound 1 has 2 UV transitions, compound 2 has 4 UV and 7 vacuum UV transitions, and compound 3 has 1 ligand-to-metal charge transition and 4 UV transitions.

Nevertheless, certain theoretically predicted transitions do not appear in the experimental spectra of the compounds. This can be attributed to their very weak intensities or the requirement of extremely high energy levels for excitation, such as in the vacuum UV region. Moreover, the type of solvent and the pH of the reaction medium can significantly influence spectral results, often leading to the broadening or merging of transition bands. Consequently, the UV–visible spectrum of a compound may display one or more wide absorption bands spanning broad wavelength ranges [62].

According to the results obtained from theory and experimental applications, the absorption bands between approximately 210 nm and 290 nm are attributed to the μ to μ* and n to μ* electronic transitions. The absorption signals in the 300–360 nm range are associated with charge transfer processes occurring from the ligand to the central metal atom [63,64]. The absorption signals appearing in the 400–700 nm range, which require less energy, are due to the interaction of visible light with the compound [65].

Table S6 highlights selected UV absorption transitions for the compounds, and the molecular orbital charge distributions corresponding to the transitions with the highest C_i_ coefficients (one example for each compound) are visualized in Figure S7. These example transitions are 99 to 102 for compound 1, 37 to 41 for compound 2, and 94 to 98 for compound 3.

The following findings are shown in Figure S7:

- In the case of HOMO - 2 (99) in compound 1, the symmetric electric charges found in both 4CP ligand molecules in the crystal structure are concentrated on only one 4CP ligand molecule with this UV transition.
- In the case of HOMO - 3 (99) in compound 2, the electric charges found in the C–H groups in the ring structure of the 4APH ionic ligand molecule are concentrated on the C–NH_2_ and N–H groups of the same ligand molecule with this UV transition.
- In the HOMO - 2 (94) state of compound 3, the electric charges on the Ni(CN)_4_ ion and a single H_2_O molecule are concentrated on only one 4APH ionic ligand molecule with this UV transition.

### 4.8. Theoretical nuclear magnetic resonance analysis of compounds 1, 2, and 3

The chemical and magnetic shift values of all elements in the theoretical and experimental nuclear magnetic resonance (NMR) spectrum of any compound, especially the ^1^H and ^13^C shift values, are strong evidence of structural changes occurring in that compound [66]. Interpreting either theoretical or experimental shift values can explain and support information about the structure of the compound. Using the crystal structure data of the 3 compounds, their NMR spectra were obtained using the Gaussian 03 program with the same basis sets used to get these data. The most popular gauge-including atomic orbitals approach was chosen for calculating the NMR spectra of the compounds.

The theoretical results obtained for compounds 1, 2, and 3 are listed in Table S7.

**Figure 9:**
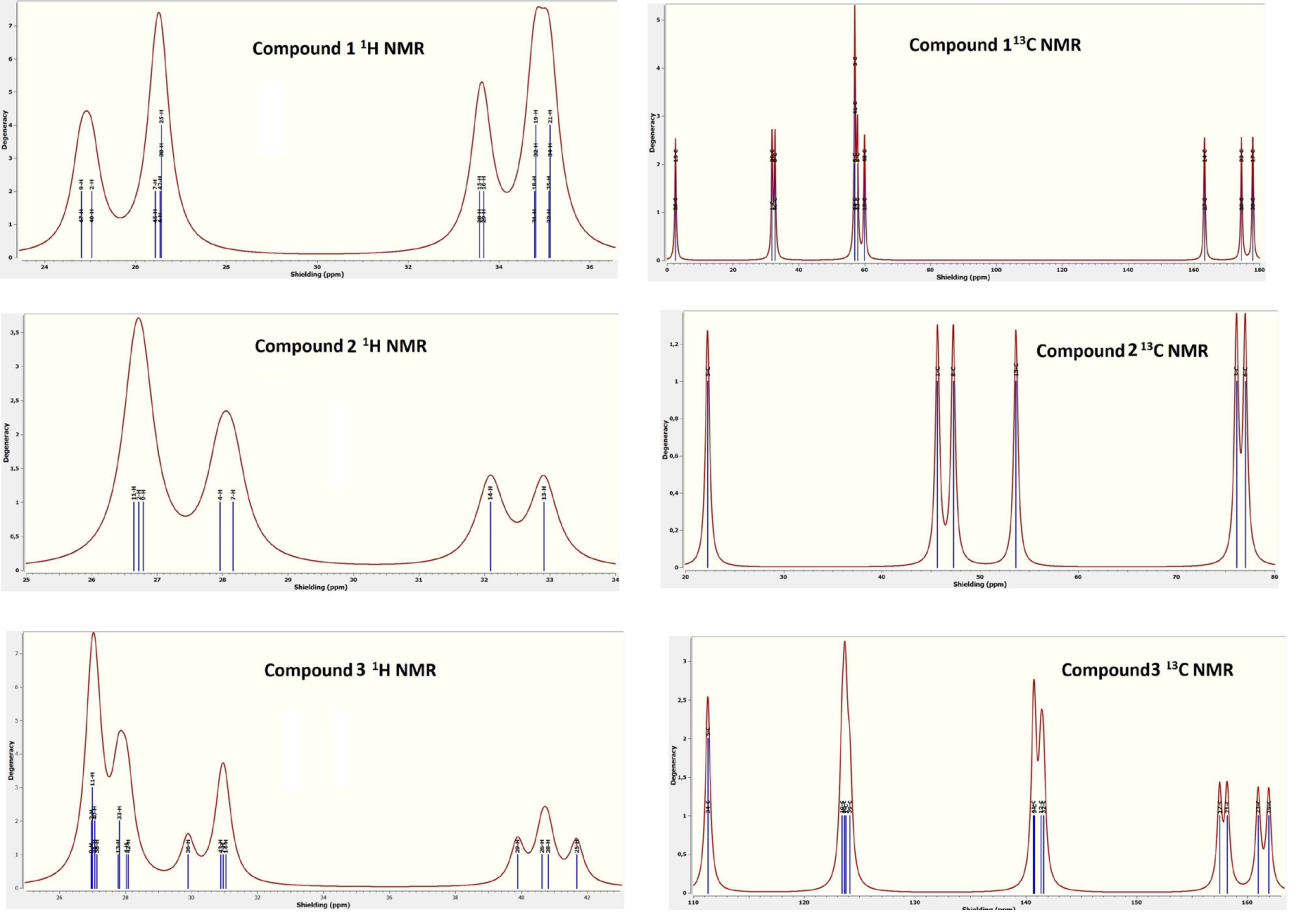
The ^1^H and ^13^C NMR spectra of compounds 1–3.

In order to provide examples of the NMR spectra of the compounds, their ^1^H and ^13^C graphs were drawn according to their average chemical shielding values (Figure 9). The following conclusions can be drawn from the NMR spectra of the compounds:

- Various elements in a compound may have positive or negative chemical shielding values depending on their location, interactions with the environment, and charge status (N, O, Ni, S, C, and H elements in compounds).
- The degeneracy number of H and C elements in compounds varies between 1–4.
- Compounds 1, 2, and 3 are formed by 21, 16, and 38 protons with different properties, respectively.
- In compound 1, the chemical shielding values of H and C elements in the chain structure SA molecule are greater than the chemical shielding values of H and C elements in the aromatic structure 4CP molecule. This is consistent with the experimental results of other researchers.
- In compound 1, the chemical shielding value of the N element in the pyridine ring in the 4CP molecule are smaller than the chemical shielding value of the N element in the C≡N group.
- In compound 1, the chemical shielding value of the O element in the carbonyl group of the SA molecule is negative, while the chemical shielding value of the O element in the OH group is positive.
- In compound 2, the H elements with the largest chemical shielding value are in the NH_2_ group, while the H element with the smallest chemical shift value is the H bonded to the N element of the pyridine ring.
- In compound 2, the N element with the largest chemical shielding value is in the NH_2_ group, while the N element with the smallest chemical shift value is in the CNS ionic group.
- In compound 2, the S element has the largest chemical shielding value, which is also in the CNS ionic group.
- The order of the positive chemical shielding values of the elements in compound 3, from largest to smallest, is as follows: the O element of the H_2_O molecule, the N element of the NH_2_ group, the N element of the pyridine ring, the C element of the C≡N group, the C element of the pyridine ring, the H element of the H_2_O molecule, and other H elements.
- Ni is the only element in compound 3 that has a negative chemical shielding value.
- In compound 3, the N elements in the 4APH ionic groups has greater chemical shielding values than the N elements in the C≡N groups, while the opposite is true for the C elements.
- The chemical shielding value of the C element in compounds generally increases as it moves away from the neighboring N element. This can be explained by the fact that the N element in compounds exerts a greater pulling force on the protons than the C element.

All values in Tables 3, 4, S6, and S7 in this study were calculated in a very short time (approximately a few milliseconds) by the ZEKA utility program developed by us and made available to all Gaussian users [67].

## 5. Conclusion

In this study, 3 new crystalline compounds were successfully synthesized and characterized using both experimental (SC-XRD, FT-IR, and elemental analysis) and theoretical (NMR, UV–visible, MEP, and Hirshfeld surface) methods. These compounds are in the space groups *P*1̄, *P*2_1_/*n*, and *C*2*/m*, respectively, and they have triclinic, monoclinic, and monoclinic crystal systems, respectively. Structural analysis confirmed the presence of strong intermolecular interactions such as hydrogen bonding and μ-type interactions, which play a crucial role in the stability and packing of the crystal lattices. The crystal structure of compound 1 consisted of C–H···O and O–H···N hydrogen bonds, as well as C–H···μ and μ···μ interactions. The crystal structure of compound 2 was composed of N–H···N and N–H···S hydrogen bonds. The crystal structure of compound 3 was composed of O–H···N and N–H···O hydrogen bonds.

Some C atoms of the 4CP molecule in compound 1 act as hydrogen bond donors to some O atoms of the SA molecule, and some other O atoms of the SA molecule act as hydrogen bond donors to some other N atoms in the 4CP molecule. As a result of these behaviors, the same shaped centrosymmetric rings are formed in compound 1, extending parallel to each other in certain directions. The N–H···N and N–H···S hydrogen bonds that form the crystal structure of compound 2 also form 2 different type centrosymmetric rings extending parallel to each other in certain directions. All Ni atoms in the [Ni(II)(CN)_4_]^2−^ ionic group in the crystal structure of compound 3 are surrounded by C atoms of the 4 cyanide groups in a square planar arrangement.

Since the void volumes of compounds 1 and 3 represent 10.26% and 11.13% of their total volumes, these compounds can serve as valuable examples for researchers studying gas storage and separation of various gas mixtures. Moreover, the theoretical data on the NLO properties of the compounds in question have considerably larger values compared to the values of many other compounds. For example, the *β*_0_ values of compounds 2 and 3 are 15.34 and 11.77 times larger than in urea, respectively. Therefore, these compounds may be interesting examples for researchers studying the NLO properties of substances.

## Supplementary Information



## Figures and Tables

**Table 1:** Selected bond lengths (Å) and bond angles (°) of compounds 1–3, °).

**Compound 1**			

N1-C1	1.335(2)	O1–C7	1.2038(18)
N1-C5	1.329(2)	O2–C7–C8	111.62(12)
N2-C6	1.140(2)	O1–C7–C8	125.40(13)
O2 -C7	1.3199(18)	N2–C6–C3	179.2(2)

**Compound 2**			

N1-C5	1.343 (2)	S1–C6	1.6379 (17)
N1-C1	1.346 (2)	N3–C6	1.159 (2)
N2-C3	1.325 (2)	N3–C6–S1	179.57 (14)

**Compound 3**			

C4—Ni1	1.8631 (19)	C3—N2	1.325 (3)
C4—N3	1.144 (2)	N1—C1	1.339 (2)
C4—Ni1—C4^i^	180.00	C4—Ni1—C1^iii^	89.26 (10)
N3—C4—Ni1	177.72 (16)	C2—C3—N2	121.64 (13)

Symmetry codes: (v) −x+1, −y+2, −z+2; (iii) x, −y+2, z.			

**Table 2:** Hydrogen bond and μ···μ interactions parameters for compounds 1–3 (Å, °).

**Compound 1**	Symmetry codes: (ii) −x+1, −y+1, −z+1; (iii) 1+x, −1+y, z; (iv) −x, −y+1, −z+1; Cg1: C1-C2-C3-C4-C5-N1			

**D—H· · ·A**	**D—H**	**H···A**	**D···A**	**D—H···A**

C4—H4···O1^ii^	0.93	2.47	3.2475 (19)	141
O2—H2A···N1	0.82	1.87	2.6873 (17)	172
C10—H10B···Cg(1)^iii^	0.97	2.93	3.8118(4)	152

**Cg(I)**	**Cg(J)**	**Cg···Cg**	**Perpendicular distance**	

Cg1	Cg1^iv^	3.7944(4)	3.3708	

**Compound 2**	Symmetry codes: (i) −x+1/2, y+1/2, −z+3/2; (ii) x−1/2, −y+3/2, z−1/2			

**D-H· · ·A**	**D-H**	**H···A**	**D···A**	**D-H···A**

N1—H1A···S1^i^	0.88(2)	2.54(2)	3.3263(15)	149.2(17)
N2—H2A···N3^ii^	0.845(18)	2.241(19)	3.065(2)	165.2(16)
N2—H2B···N3	0.85(2)	2.20(2)	3.024(2)	165.8(18)

**Compound 3**	Symmetry codes: (vi) −*x*+1, *y*, −*z*+1; (vii) −*x*+1, −*y*+1, −*z*+2			

**D-H· · ·A**	**D-H**	**H···A**	**D···A**	**D-H···A**

N2—H3···O1^vii^	0.883 (17)	2.048 (18)	2.917 (2)	168.1 (17)
O1—H4···N3^vi^	0.79 (2)	2.07 (2)	2.856 (2)	171 (2)

**Table 3:** The HOMO, LUMO, and chemical efficiency values in eV units of compounds.

Chemical efficiency values	1	2	3	

	6–311G(d,p)	6–311G(d,p)	6–311G(d,p)	LanL2MB

E_HOMO_ (−I)	−7.198	−3.632	−5.869	−2.150
E_LUMO_ (−A)	−2.897	−3.013	−5.736	−1.582
ΔE = E_LUMO_ − E_HOMO_	4.301	0.619	0.133	0.568
χ	5.048	3.323	−5.803	1.866
μ	−5.048	−3.323	5.803	−1.866
η	2.151	0.310	−0.067	0.284
S (eV)^−1^	0.233	1.616	−7.499	1.761
ω	5.925	17.839	−252.54	6.130

**Table 4:** The electric dipole moment, anisotropies of polarizability, the mean polarizability, the first- and second-order static hyperpolarizabilities of compounds 1–3.

Parameters	1	2	3
μ (D)	0.6176	25.4790	17.0700
Δα (esu)	1.3339 × 10^−23^	5.1250 × 10^−24^	4.3946 × 10^−23^
α_0_ (esu)	−2.6058 × 10^−23^	−1.0761 × 10^−23^	−6.9354 × 10^−24^
β_0_ (esu)	2.8441 × 10^−31^	5.6758 × 10^−30^	4.3536 × 10^−30^
γ (esu)	−1.261 × 10^−35^	−9.5498 × 10^−37^	−2.1688 × 10^−36^

**Table 5:** Thermodynamic parameters of compounds 1–3.

Thermodynamic parameters	Components	1	2	3
E (kcal/mol)	Electronic	0	0	0
	Translational	0.889	0.889	0.889
	Rotational	0.889	0.889	0.889
	Vibrational	257,543	83,619	222,237

	Total	259,321	85,396	224,014

Heat capacity at constant volume C_V_ (cal/mol-Kelvin)	Electronic	0	0	0
	Translational	2.981	2.981	2.981
	Rotational	2.981	2.981	2.981
	Vibrational	59,391	17,073	61,666

	Total	65,352	23,034	67,627

Entropy S (cal/mol-Kelvi)	Electronic	0	0	0
	Translational	43,714	40,986	43.76
	Rotational	37,479	31,193	36,949
	Vibrational	51,597	14,532	79,344

	Total	132,79	86,711	160,054

Zero-point vibrational energy E_v0_	(Joules/mol)	1,042,433	339,833	884,606
	(kcal/mol)	249,147	81,2221	211,426

Rotational constants (GHz)	A	1.41642	4.71423	0.40874
	B	0.02121	0.28051	0.05342
	C	0.02091	0.26557	0.04904

**Table 6:** Relative proportions of different intermolecular interactions observed in the 2D fingerprint maps of the compounds, arranged in descending order based on their contribution rates.

1		2		3	

Interactions	Contrib. (%)	Interactions	Contrib. (%)	Interactions	Contrib. (%)
H···H	35.8	H···H	25.4	H···H	31.4
H···N/N···H	20.5	C···H/H···C	24.5	H···N/N···H	31.1
H···O/O···H	17.2	H···S/S···H	23.9	C···H/H···C	12.8
C···H/H···C	15.4	H···N/N···H	15.1	C···C	9.4
C···C	2.4	C···N/N···C	4.9	H···O/O···H	7.6
C···N/N···C	2.2	C···S/S···C	3.9	C···N/N···C	4.0
C···O/O···C	1.9	S···N/N···S	1.9	C···Ni/Ni···C	3.0
O···O	0.2	N···N	0.3		
		C···C	0.1		
