# Supplementary material for: Synthesis, characterization, and theoretical study of new cocrystals and charge-transfer compounds
Source: Turk J Chem. 2025 Aug 19;49(6):717–35. doi: 10.55730/1300-0527.3766 (PMC12779054; doi:10.55730/1300-0527.3766)
Supplement: Supplementary file 1 [file 49-6-717-supp.docx]

**Synthesis, characterization, and theoretical study of new cocrystals and charge-transfer compounds**

Zarife Sibel ŞAHİN^1,*^, Zeki KARTAL^2^

^1^Department of Energy Systems Engineering, Faculty of Engineering and Architecture, Sinop University, Sinop, Turkiye

^2^Retired Professor of Atomic and Molecular Physics, Kütahya Dumlupınar University, Kütahya, Turkiye

^*^ Correspondence: [zarifesibel@sinop.edu.tr](mailto:zarifesibel@sinop.edu.tr)

Zarife Sibel ŞAHİN: https://orcid.org/ 0000-0003-2745-7871

Zeki KARTAL: https://orcid.org/0000-0001-9739-0858

**Supplementary materials**

**
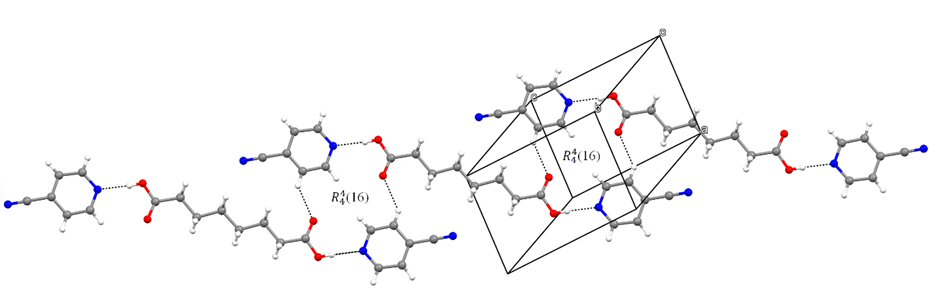
**

**Figure S1.** A portion of the crystal structure of compound 1 showing the formation of $R_{4}^{4}$(16) rings edge fused along [111].


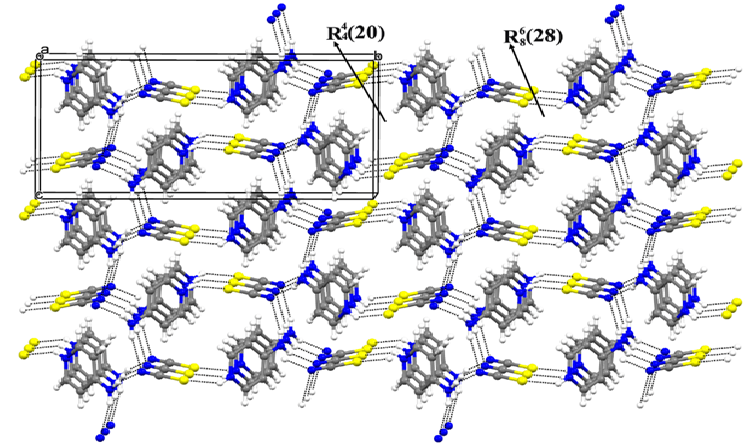


**Figure S2.** A portion of the crystal structure of compound 2 illustrating the formation of chain edge-fused $R_{8}^{6}$(28) and $R_{4}^{4}(20)$ rings.


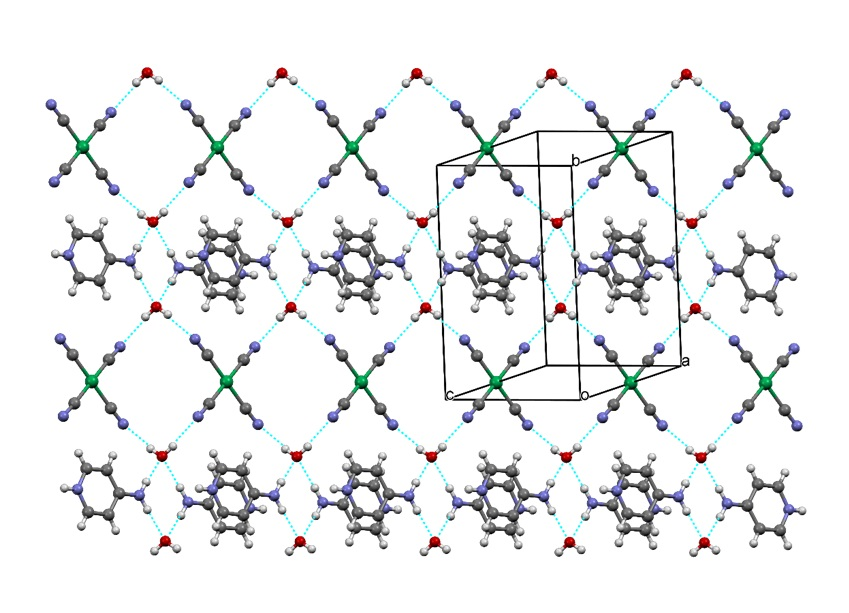


**Figure S3.** An infinite 3D supramolecular network.


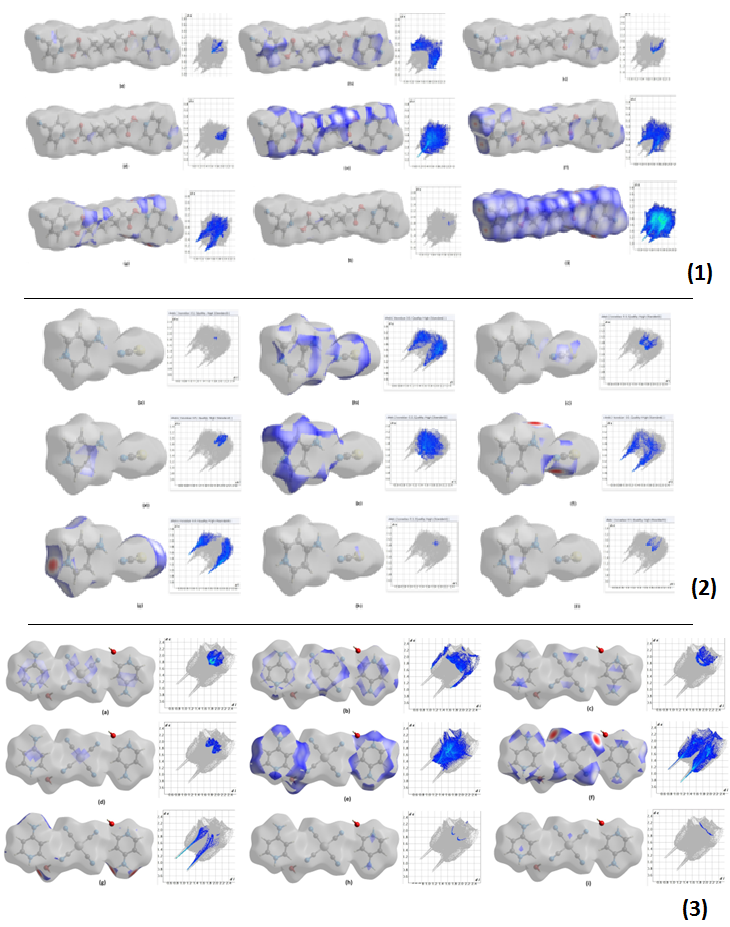


**Figure S4.** 2D fingerprint plots were generated to illustrate these interactions based on surface projections of compounds 1–3.


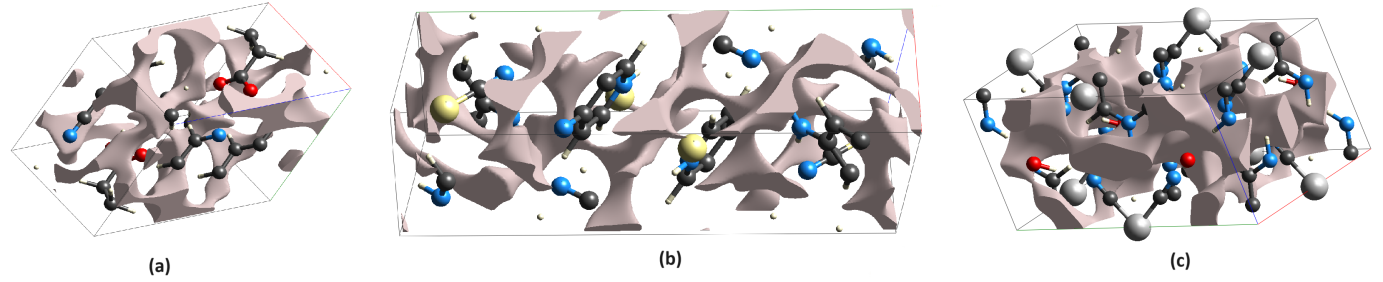


**Figure S5**. 3D views of the voids formed depending on the electron arrangement in the structure for (a) compound 1, (b) compound 2, and (c) compound 3.


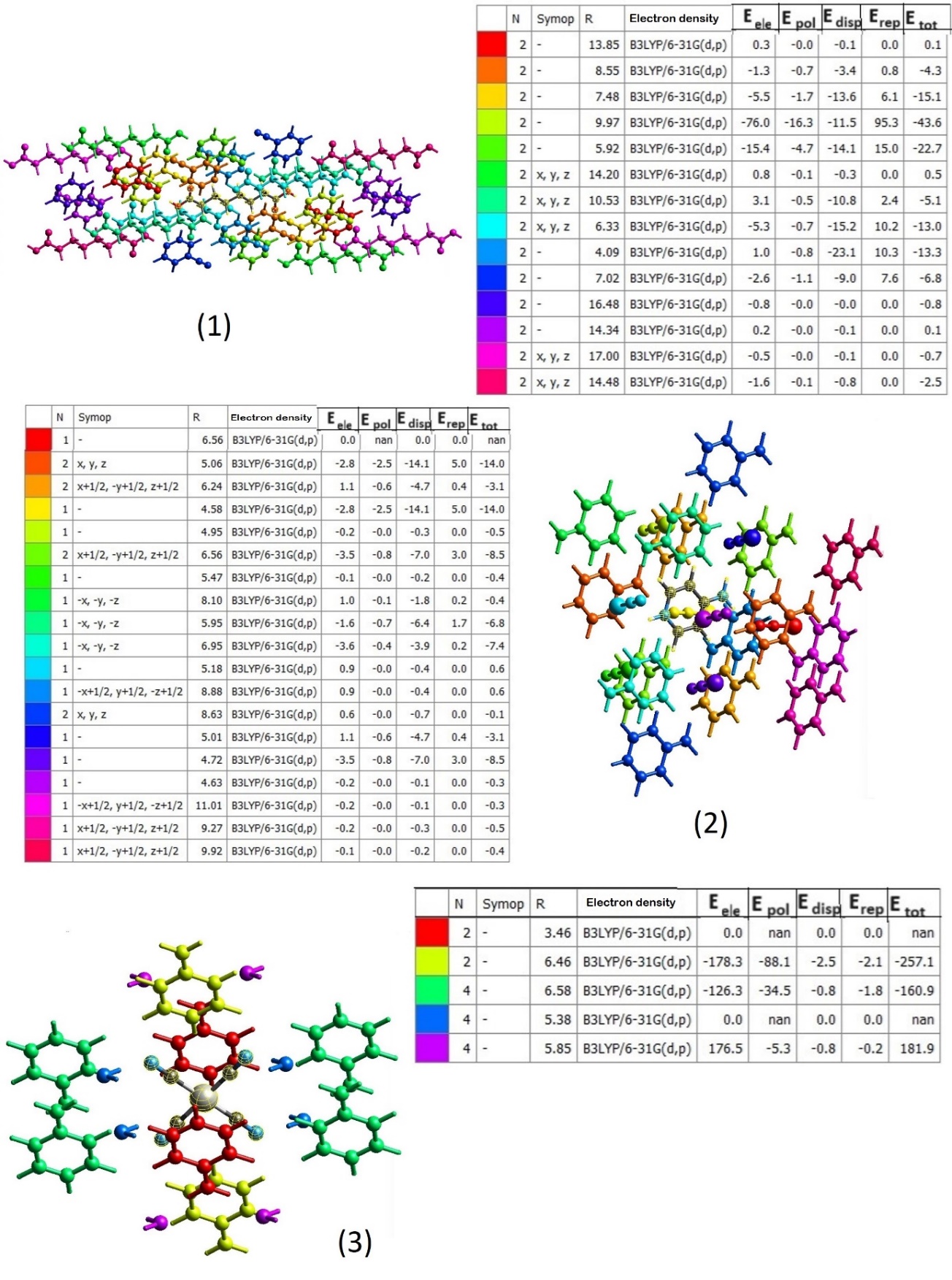


**Figure S6**. Energy values of the components of the total interaction energies of compounds 1–3. In the tables, N denotes the number of molecular pairs interacting with the reference molecule, Symop refers to the symmetry operations applied to the reference molecule, R indicates the distance (in Å) between the center of mass of the reference molecule and those of its neighboring molecules, and E represents the individual components of the calculated interaction energies.


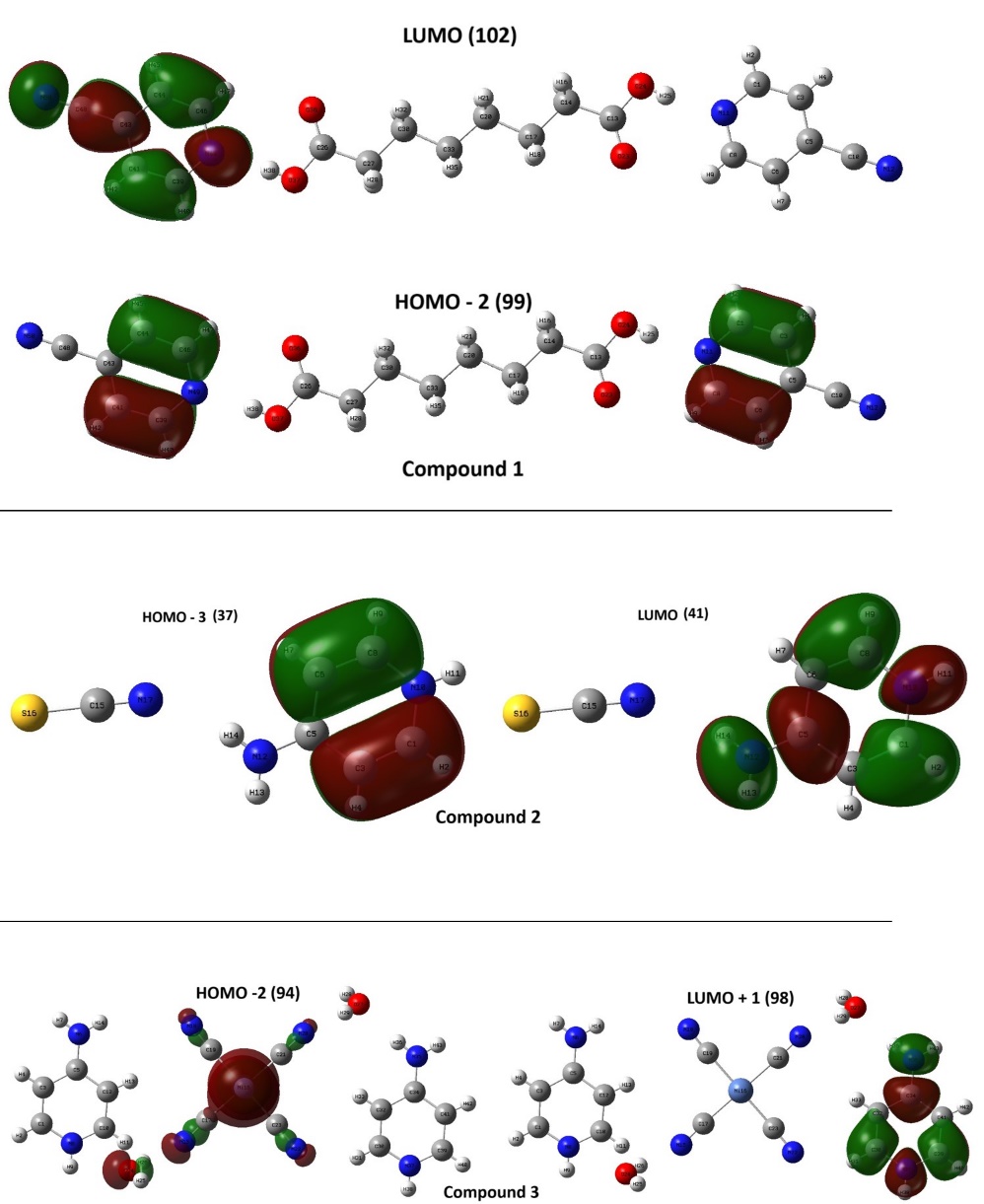


**Figure S7.** The charge distribution graphs of the UV transitions with the highest C_i_ coefficients of compounds 1, 2 and 3.

**Table S1.** Elemental analysis data for the compounds.

| Compounds; M_r_ (g) | Elemental analysis, (calculated) (%)/found (%) | | | | |
| --- | --- | --- | --- | --- | --- |
|  | C | H | N | S | Ni |
| [(C_8_H_14_O_4_)(C_6_H_4_N_2_)_2_];  382.41 | (62.82)  62.79 | (5.80)  5.83 | (14.65)  14.71 | (-)  - | (-)  - |
| [(C_5_H_7_N_2_)^+^·(NCS)^-^];  153.20 | (47.04)  46.96 | (4.61)  4.64 | (27.43)  27.51 | (20.93)  20.98 | (-)  - |
| [Ni(CN)_4_.2(C_5_H_7_N_2_).2(H_2_O)]; 389.04 | (43.22)  43.08 | (4.66)  4.61 | (28.80) 28.91 | (-)  - | (15.09) 15.12 |

**Table S2.** Crystallographic data of compounds 1-3.

| Crystal data | **1** | **2** | **3** |
| --- | --- | --- | --- |
| Empirical formula | C_20_H_22_N_4_O_4_ | C_6_H_7_N_3_S | C_14_H_18_N_8_O_2_Ni |
| Formula weight | 382.41 | 153.21 | 389.07 |
| Crystal system | Triclinic | Monoclinic | Monoclinic |
| Space group | *P*$\bar{1}$ | *P*2_1_/*n* | *C*2/*m* |
| *a (*Å) | 6.3263 (6) | 5.0581 (5) | 10.4345 (13) |
| *b (*Å) | 8.5643 (8) | 17.3940 (17) | 12.6008 (16) |
| *c (*Å) | 9.5987 (10) | 8.6270 (8) | 7.3076 (10) |
| *α (*º) | 79.856 (4) | 90.000 | 90.000 |
| *β (*º) | 79.921 (5) | 95.412 (3) | 108.091 (5) |
| *γ (*º) | 83.893 (4) | 90.000 | 90.000 |
| *V (*Å^3^) | 502.50 (9) | 755.63 (13) | 913.3 (2) |
| Z | 1 | 4 | 2 |
| *D*_c_ (g cm^-3^) | 1.264 | 1.347 | 1.415 |
| μ (mm^-1^) | 0.09 | 0.35 | 1.09 |
| θ range (º) | 3.0–28.0 | 2.3–26.9 | 2.6–28.4 |
| Measured refls. | 26630 | 20758 | 8070 |
| Independent refls. | 2422 | 1609 | 1200 |
| *R*_int_ | 0.063 | 0.028 | 0.050 |
| S | 1.07 | 1.10 | 1.08 |
| R1/wR2 | 0.052/0.159 | 0.033/0.076 | 0.036/0.079 |
| T_max_/T_min_ | 0.642/0.746 | 0.667/0.745 | 0.437, 0.746 |
| CCDC | 2334512 | 2334585 | 2374519 |

**Table S3**. Vibration frequencies in the free states of the ligands used in the preparation of compounds and in the compounds.

| Assignment | 4CP ^a^ | SA ^b^ | **1** | 4AP ^c^ | KSCN^d^ | **2** | 4AP ^c^ | K_2_Ni(CN)_4_·H_2_O ^d^ | **3** |
| --- | --- | --- | --- | --- | --- | --- | --- | --- | --- |
| ν(O-H) Free H_2_O | - | - | - | - | - | - | - | - | 3604 w |
| ν_as_(O-H) water | - | - | - | - | - | - | - | 3469 w | **-** |
| ν_s_(O-H) water | - | - | - | - | - | - | - | 3245 w | **-** |
| ν(O-H) carboxy. | - | 3416 w | 2668 w | - | - | - | - | - | - |
| ν_as_(NH_2_) | - | - | - | 3430 s | - | 3458 s | 3430 s | - | 3384 w |
| ν(N-H) | - | - | - | - | - | 3437 s | - | - | 3334 s |
| ν_s_(NH_2_) | - | - | - | 3303 w | - | 3349 s | 3303 w | - | 3235 m |
| ν(C-H) aromatic | 3089 w | - | 3153 w  3113 w | 3100 w | - | 3219 m | 3100 w | - | 3160 m |
| ν(C-H) aromatic | 3023 w | - | 3088 m  3041 w | 3079 w | - | 3068 w | 3079 w | - | 3053 m |
| ν(C-H) aliphatic | - | 2943 s | 2943 s | - | - | - | - | - | - |
| ν(COH) | - | 2869 m | 2866 w | - | - | - | - | - | - |
| δ(O-H) carboxylic | - | 2530 w | 2765 w | - | - | - | - | - | - |
| ν(C≡N) | 2235 m | - | 2240 m | - | 2036 s | 2104 s  2071 s  2023 w | - | 2121 s | 2123 s |
| ν(C=O) carboxy. | - | 1683 s | 1709 s | - | - | - | - | - | - |
| δ(OH) water | - | - | - | - | - | - | - | 1652 s | 1650 s |
| δ(NH_2_) | - | - | - | 1649 m | - | 1685 w | 1649 m | - | 1673 s |
| ν(C=C) | 1588 m | - | 1600 s | 1585 s | - | 1624 s | 1585 s | - | 1650 s |
| ν(C-C) | 1541 m |  | 1547 m | 1429 w | - | 1449 m | 1429 w | - | 1402 m |
| ν(C-N) | 1486 m | - | 1499 m | 1267 m | - | 1280 m | 1267 m | - | 1241 w |
| δ(CH_2_) | - | 1466 w | 1465 w | - | - | - | - | - | - |
| ν(C-N) | 1413 m | - | 1410 s | - | - | - | - | - | - |
| δ(C=O) carboxy., δ(HCH) | - | 1332 s  1254 s  1190 s | 1332 s  1240 s  1179 s | - | - | - | - | - | - |
| ν_ring_ (2.) | 1079 m | - | 1071w | - | - | - |  | - | - |
| ω(CH_2_), δ(C-O) | - | 1059w | 1064 s | - | - | - | - | - | - |
| ν_ring_ (1.) | 982 m | - | 1007 s | 976 s | - | 1013 s | 976 s | - | 997 s |
| ν(C-S) | - | - | - | - | 740 m | 824 s | - | - | - |
| ν(Ni-CN) | - | - | - | - | - | - | - | 544 w | 530 w |
| δ(NCS) | - | - | - | - | 474 s | 529 s | - | - | - |
| π(Ni-CN) | - | - | - | - | - | - | - | 442 w | 496 s |
| δ(Ni-CN) | - | - | - | - | - | - | - | 420 w | 414 s |

ν: stretching; δ: in plane bending; π: out of plane bending; ω: wagging; s: strong; m: medium; w: weak; **^a^** taken from Macrae et al. [36], **^b^** taken from Kartal and Şahin [21].

**Table S4**. Mulliken electric charges of the atoms in compounds 1–3 and their values ​​in terms of free electron charge (e).

| **Compound 1** | | | | | | | | |
| --- | --- | --- | --- | --- | --- | --- | --- | --- |
| **Atom** | **ACVFL** | **ACVC** | **Atom** | **ACVFL** | **ACVC** | **Atom** | **ACVFL** | **ACVC** |
| C1 | 0.185957 | 0.099877 | H9A | 0.142383 | 0.171837 | H10B | 0.141712 | 0.159087 |
| H1 | 0.023729 | 0.198678 | H9B | 0.15869 | 0.191049 | O1^i^ | -0.30536 | -0.42514 |
| C2 | -0.1224 | -0.16006 | C10 | -0.23221 | -0.33283 | O2^i^ | -0.21499 | -0.56903 |
| H2 | 0.060696 | 0.203404 | H10A | 0.140167 | 0.160398 | H2A^i^ | 0.26926 | 0.41346 |
| C3 | 0.212041 | 0.125705 | H10B | 0.135592 | 0.173881 | C5^i^ | 0.18596 | 0.09995 |
| C4 | -0.12165 | -0.1438 | O1 | -0.25318 | -0.42502 | H5^i^ | 0.023729 | 0.19919 |
| H4 | 0.074322 | 0.206395 | O2 | -0.1496 | -0.56927 | C4^i^ | -0.1224 | -0.15988 |
| C5 | 0.192347 | 0.079094 | H2A | 0.240284 | 0.413334 | H4^i^ | 0.060696 | 0.203937 |
| H5 | 0.038906 | 0.248688 | C7 | 0.036922 | 0.521698 | C3^i^ | 0.212041 | 0.125701 |
| C6 | 0.136608 | -0.21773 | C8 | -0.14066 | -0.46139 | C6^i^ | -0.12165 | -0.14398 |
| N1 | -0.36829 | -0.5239 | H8A | 0.187923 | 0.207189 | H2^i^ | 0.074322 | 0.20588 |
| N2 | -0.31227 | -0.0685 | H8B | 0.169527 | 0.21938 | C2^i^ | 0.192347 | 0.07899 |
| C7 | -0.46826 | 0.521449 | C9 | -0.35082 | -0.31916 | H1^i^ | 0.038906 | 0.248284 |
| C8 | -0.32338 | -0.46108 | H9A | 0.154158 | 0.186726 | C1^i^ | 0.136608 | -0.21773 |
| H8A | 0.183773 | 0.221749 | H9B | 0.164792 | 0.178489 | N1^i^ | -0.36829 | -0.52395 |
| H8B | 0.160461 | 0.206576 | C10 | -0.27269 | -0.33276 | N2^i^ | -0.31227 | -0.06852 |
| C9 | -0.04779 | -0.31918 | H10A | 0.14608 | 0.172796 |  |  |  |
| Symmetry code: (i) -x, 2-y, 2-z | | | | | | | | |
| **Compound 2** | | | | | | | | |
| **Atom** | **ACVFL** | **ACVC** | **Atom** | **ACVFL** | **ACVC** | **Atom** | **ACVFL** | **ACVC** |
| C1 | -0.339235 | 0.182705 | H4 | 0.107513 | 0.267343 | H2A | 0.233609 | 0.356867 |
| H1 | 0.129635 | 0.240808 | C5 | -0.339235 | 0.190058 | H2B | 0.233609 | 0.387064 |
| C2 | 0.253458 | -0.29816 | H5 | 0.129635 | 0.247489 | C6 | 0.096503 | -0.23122 |
| H2 | 0.107513 | 0.237178 | N1 | -0.112994 | -0.67646 | S1 | 0.107499 | -0.23649 |
| C3 | -0.293325 | 0.422293 | H1A | - | 0.358431 | N3 | -0.204003 | -0.39235 |
| C4 | 0.253458 | -0.25616 | N2 | -0.363642 | -0.7994 |  |  |  |
| **Compound 3** | | | | | | | | |
| **Atom** | **ACVFL** | **ACVC** | **Atom** | **ACVFL** | **ACVC** | **Atom** | **ACVFL** | **ACVC** |
| C1 | 0,310923 | 0,267627 | Ni1 | 2,183421 | -0,090983 | H1^ii^ | 0.015475 | 0.180884 |
| H1 | 0,015475 | 0,107014 | C4 | -0,707764 | 0,300629 | C2^ii^ | -0.254572 | -0.291715 |
| C2 | -0,254572 | -0,2859 | N3^iv^ | -0,337463 | -0,591525 | H2^ii^ | 0.029941 | 0.134226 |
| H2 | 0,029941 | 0,083524 | C4^iv^ | -0,708138 | 0,116542 | C3^ii^ | 0.657298 | 0.82504 |
| C3 | 0,657298 | 0,823835 | N3^v^ | -0,337221 | -0,67594 | N2^ii^ | -0.704055 | -0.769413 |
| N2 | -0,704055 | -0,734464 | C4^v^ | -0,709188 | 0,212215 | H3^ii^ | 0.199996 | 0.356279 |
| H3A | 0,199996 | 0,255071 | N3^iii^ | -0,337316 | -0,702929 | N1^ii^ | -0.556768 | -0.442579 |
| N1 | -0,556768 | -0,414606 | C4^iii^ | -0,708784 | 0,204251 | H1A^ii^ | 0 | 0.273931 |
| H1A | 0 | 0,28208 | O1 | -0,714341 | -0,726339 | C1^iii^ | 0.310923 | 0.268312 |
| C1^i^ | 0,310923 | 0,336997 | H4 | 0,357171 | 0,317839 | H1^iii^ | 0.015475 | 0.103998 |
| H1^i^ | 0,015475 | 0,182776 | H4^i^ | 0,357171 | 0,395203 | C2^iii^ | -0.254572 | -0.29349 |
| C2^i^ | -0,254572 | -0,288087 | O1^v^ | -0.714341 | -0.766539 | H2^iii^ | 0.029941 | 0.082008 |
| H2^i^ | 0,029941 | 0,135542 | H4^v^ | 0.357171 | 0.320312 | H3^iii^ | 0.199996 | 0.252242 |
| H3^i^ | 0,199996 | 0,28019 | H4^ii^ | 0.357171 | 0.320312 |  |  |  |
| N3 | -0,337546 | -0,795252 | C1^ii^ | 0.310923 | 0.341625 |  |  |  |
| Symmetry codes: (*i*) x, −y+1, z; (*ii*) x, y+1, z; (*iii*) x, −y+2, z; (*iv*) 1−x, y, −z+2; (*v*) 1−x, −y+2, −z+2 | | | | | | | | |

ACVFL: atomic charge value in free ligand, ACVC: atomic charge value in the compound.

**Table S5**. General properties of voids in unit cells of compounds 1–3.

| Compounds | Volume (Å³) | RVVTV (%) | Void area (Å²) | Globularity | Asphericity |
| --- | --- | --- | --- | --- | --- |
| **1** | 51.57 | 10.26 | 209.23 | 0.320 | 0.071 |
| **2** | 62.01 | 08.21 | 283.34 | 0.267 | 0.363 |
| **3** | 101.62 | 11.13 | 399.12 | 0.264 | 0.094 |

RVVTV (%): ratio of void volume to total volume (%).

**Table S6.** Theoretically calculated absorption wavelength (λ), excitation energy values, ​​​​and oscillator strengths (f) for electronic transitions in the UV–visible region of compounds 1–3 in water.

| Compounds | Exc. States | Exc. energy (eV) | W. length (nm) | Osc. strengths | Assignments | | Coefficients  C_i_ |
| --- | --- | --- | --- | --- | --- | --- | --- |
|  |  |  |  |  | From → to | |  |
| **1** | 1 | 4.5752 | 270.99 | 0.0022 | 101 →102 | | 0.35297 |
|  | 2 | 5.0403 | 245.99 | 0.0537 | 99 →102 | | 0.42547 |
| **2** | 1 | 5.2943 | 234.18 | 0.0311 | 40 → 43 | 0.60991 | |
|  | 2 | 5.2998 | 233.94 | 0.0365 | 39 → 43 | 0.60626 | |
|  | 3 | 5.3711 | 230.83 | 0.3522 | 38 → 41 | 0.60937 | |
|  | 4 | 5.9019 | 210.07 | 0.0065 | 40 → 48 | 0.30511 | |
|  | 5 | 5.9284 | 209.14 | 0.0153 | 39 → 44 | 0.44050 | |
|  | 6 | 5.9374 | 208.82 | 0.0094 | 40 → 44 | 0.36001 | |
|  | 7 | 6.2667 | 197.84 | 0.2529 | 37 → 41 | 0.61766 | |
|  | 8 | 6.3377 | 195.63 | 0.0783 | 39 → 45 | 0.44512 | |
|  | 9 | 6.3555 | 195.08 | 0.0099 | 40 → 46 | 0.43463 | |
|  | 10 | 6.4032 | 193.63 | 0.1327 | 39 → 46 | 0.40721 | |
|  | 11 | 6.6465 | 186.54 | 0.1281 | 39 → 48 | 0.33577 | |
| **3** | 1 | 3.8337 | 323.40 | 0.0046 | 96 →100 | 0.63710 | |
|  | 2 | 4.4894 | 276.17 | 0.0018 | 95 →100 | 0.53491 | |
|  | 3 | 4.5040 | 275.28 | 0.0010 | 95 →101 | 0.61746 | |
|  | 4 | 4.5855 | 270.38 | 0.0010 | 94 → 98 | 0.69440 | |
|  | 5 | 4.6296 | 267.81 | 0.0010 | 91 → 97 | 0.62552 | |

**Table S7**. The NMR theoretical isotropic and anisotropic chemical and magnetic shielding values calculated for compounds 1–3 in water at room temperature.

| Atoms | Degeneracy | Chemical and Magnetic shielding values (ppm) | | | Atoms | | Degeneracy | | Chemical and Magnetic shielding values (ppm) | | | | |
| --- | --- | --- | --- | --- | --- | --- | --- | --- | --- | --- | --- | --- | --- |
|  |  | Isotropic | | Anisotropic |  |  |  |  | Isotropic | | | Anisotropic | |
|  |  | Average | Shielding value corrected to TMS |  |  |  |  |  | Average | Shielding value corrected to TMS | |  |  |
| **Compound 1** | | | | | | **Compound 3** | | | | | | | |
| N1; N1^i^ | 2 | -77.4686 | - | 582.0642; 582.0335 | Ni1 | | 1 | | -2586.7388 | - | | 1827.9694 | |
| O1^i^; O1 | 2 | -55.5268 | - | 548.0302; 547.9990 | N3^iv^ | | 1 | | 18.4187 | - | | 404.4922 | |
| N2^i^; N2 | 2 | -20.9669 | - | 404.8143; 404.8767 | N3^iii^ | | 1 | | 19.0846 | - | | 404.1132 | |
| C7^i^; C7 | 2 | 2.5300 | 179.9356 | 110.6551; 110.6303 | H1A; H1; H1^i^ | | 3 | | 27.0021 | 4.8800 | | 10.5859; 3.8035; 3.7475 | |
| H1^i^; H5 | 2 | 24.8471 | 7.0350 | 12.6492; 12.6487 | H1^ii^; H2 | | 2 | | 27.0939 | 4.7882 | | 4.5607; 5.3910 | |
| 40; 2 | 2 | 25.0739 | 6.8082 | 10.9427; 10.9453 | H1A^ii^ | | 1 | | 27.1650 | 4.7171 | | 11.3373 | |
| H2^i^; H4 | 2 | 26.4604 | 5.4218 | 10.4524; 10.4497 | N3^v^ | | 1 | | 27.2534 | - | | 394.4694 | |
| H4i; H2; H2A^i^; H2A | 4 | 26.5820 | 5.3001 | 9.6859; 9.6866; 25.7544; 25.7555 | N3 | | 1 | | 27.3236 | - | | 394.5214 | |
| C1; C5^i^ | 2 | 31.8678 | 150.5979 | 205.1792; 205.1587 | H2^i^; C2^ii^ | | 2 | | 27.8204 | 4.0617 | | 5.6677; 5.4956 | |
| C2^i^; C5 | 2 | 32.7743 | 149.6913 | 208.3641; 208.3544 | H2 | | 1 | | 28.0483 | 3.8338 | | 5.3910 | |
| H8A^i^; H8A | 2 | 33.5398 | -1.6577 | 6.4264; 6.4265 | H2^iii^ | | 1 | | 28.1013 | 3.7808 | | 5.7788 | |
| H8B^i^; H8B | 2 | 33.6326 | -1.7505 | 6.8397; 6.8402 | H3^ii^ | | 1 | | 29.9086 | 1.9735 | | 23.0404 | |
| H9A^i^; H9A; H9B^i^; H9B | 4 | 34.7525 | -2.8704 | 6.5594; 6.5588; 6.4658; 6.4662 | H3^iii^ | | 1 | | 30.8971 | 0.9850 | | 16.0191 | |
| H10B^i^; H10B; H10A^i^; H10A | 4 | 35.0636 | -3.1815 | 5.6887; 5.6887; 5.8884; 5.8883 | H3 | | 1 | | 30.9689 | 0.9132 | | 17.3244 | |
| C6^i^; C4; C4^i^; C2 | 4 | 57.0092 | 125.4565 | 205.0740; 205.0513; 204.6876; 204.6891 | H3^i^ | | 1 | | 31.0493 | 0.8328 | | 16.3431 | |
| C3^i^; C3 | 2 | 57.8151 | 124.6505 | 166.5969; 166.5523 | H4^ii^ | | 1 | | 39.8542 | -7.9721 | | 33.1446 | |
| C6; C1^i^ | 2 | 59.9681 | 122.4975 | 343.5704; 343.5635 | H4^i^ | | 1 | | 40.5973 | -8.7152 | | 32.6554 | |
| O2^i^; O2 | 2 | 136.5687 | - | 150.3062; 150.3564 | H4^v^ | | 1 | | 40.7827 | -8.9006 | | 27.4667 | |
| C8^i^; C8 | 2 | 163.2417 | 19.2239 | 60.3515; 60.3550 | H4 | | 1 | | 41.6419 | -9.7598 | | 27.7890 | |
| C10^i^; C10 | 2 | 174.4890 | 7.9767 | 40.3303; 40.3291 | C3^ii^; C3 | | 2 | | 111.2826 | 71.1830 | | 145.0106; 144.8900 | |
| C9^i^; C9 | 2 | 177.9147 | 4.5510 | 34.1464; 34.1461 | C1^i^ | | 1 | | 123.3134 | 59.1522 | | 132.0055 | |
| **Compound 2** | | | | | | C1^ii^ | | 1 | 123.5421 | | 58.9235 | | 130.9552 |
| C3 | 1 | 20.1622 | 162.3034 | 212.9816 | C1 | | 1 | | 123.6703 | 58.7953 | | 131.0293 | |
| H1A | 1 | 26.2784 | 5.6037 | 5.2996 | C1^iii^ | | 1 | | 124.0354 | 58.4302 | | 130.5657 | |
| H1 | 1 | 26.4928 | 5.3893 | 6.7229 | C2^iii^ | | 1 | | 140.5114 | 41.9542 | | 130.1068 | |
| H5 | 1 | 26.5561 | 5.3260 | 6.9611 | C2 | | 1 | | 140.5915 | 41.8741 | | 130.8112 | |
| H2 | 1 | 27.6679 | 4.2142 | 8.1085 | C2^i^ | | 1 | | 141.1750 | 41.2906 | | 129.6856 | |
| H3 | 1 | 27.8076 | 4.0745 | 8.4217 | C2^ii^ | | 1 | | 141.4054 | 41.0602 | | 128.8266 | |
| H2B | 1 | 31.5921 | 0.2900 | 19.5286 | C4 | | 1 | | 157.2165 | 25.2491 | | 195.3953 | |
| H2A | 1 | 32.5481 | -0.6660 | 8.8607 | C4^v^ | | 1 | | 157.8921 | 24.5735 | | 194.5823 | |
| N3 | 1 | 40.9136 | - | 461.3638 | C4^iii^ | | 1 | | 160.6767 | 21.7889 | | 190.2298 | |
| C1 | 1 | 44.8297 | 137.6359 | 188.5759 | C4^iv^ | | 1 | | 161.6263 | 20.8393 | | 188.7025 | |
| C5 | 1 | 46.2372 | 136.2284 | 187.5499 | N1 | | 1 | | 207.9435 | - | | 142.1206 | |
| C6 | 1 | 46.6949 | 135.7707 | 367.2538 | N1^ii^ | | 1 | | 208.9679 | - | | 140.1005 | |
| C2 | 1 | 75.5923 | 106.8733 | 164.1584 | N2^ii^ | | 1 | | 253.0385 | - | | 40.2423 | |
| C3 | 1 | 76.1971 | 106.2685 | 164.6129 | N2 | | 1 | | 255.5806 | - | | 35.4744 | |
| N1 | 1 | 101.1524 | - | 227.4076 | O1 | | 1 | | 431.0153 | - | | 19.1125 | |
| N2 | 1 | 179.3116 | - | 65.2685 | O1^v^ | | 1 | | 433.2705 | - | | 19.5665 | |
| S1 | 1 | 800.4815 | - | 393.7182 |  | |  | |  |  | |  | |
